# Impact of Lignin Type on Yield and Fiber Morphology in Biobased Carbon Fiber Precursors

**DOI:** 10.1021/acsomega.5c12442

**Published:** 2026-03-25

**Authors:** Jenny Bengtsson, Leandro Cid Gomes, Feryal Guerroudj, Hanna Ulmefors, Annika I. Altskär, Michael Hummel, Diana Bernin

**Affiliations:** † Department of Chemistry and Chemical Engineering, 11248 Chalmers University of Technology, 412 96 Gothenburg, Sweden; ‡ Department of Polymer, Fiber and Composites, 388792 RISE Research Institutes of Sweden, 431 53 Mölndal, Sweden; § Chalmers Industriteknik, 412 58 Gothenburg, Sweden; ∥ Department Agriculture and Food, RISE, Research Institutes of Sweden, 412 76 Gothenburg, Sweden; ⊥ Department of Bioproducts and Biosystems, 174277 Aalto University, P.O. Box 16300, 0076 Aalto, Finland

## Abstract

The quest for sustainable materials has stimulated extensive research into biobased carbon fibers, with lignin–cellulose composite fibers emerging as promising candidates. However, challenges persist, for example, the leaching of lignin during fiber spinning, which limits the process efficiency and carbon fiber yield. Thus, this work aims to understand the causes and the impact of lignin leaching. The lignin was varied in terms of source, extraction technique, and molecular weight, and the lignin yield, fiber morphology, and mechanical performance of cellulose–lignin composite fibers were elucidated. The results demonstrated that the lignin’s functional groups significantly influence lignin yield in spun fibers, and a high molecular weight is beneficial. An expressive decrease in lignin yield was observed for lignin with a higher content of polar functional groups such as carboxylic acid groups and a lower content of condensed lignin moieties. Furthermore, leaching of lignin was also associated with the observed defects and deteriorated mechanical properties of the precursor fiber. Addressing these challenges is thus critical for maximizing the potential of biobased carbon fibers, emphasizing the need to mitigate lignin leaching and optimize fiber processing parameters for enhanced fiber quality and performance.

## Graphical abstract

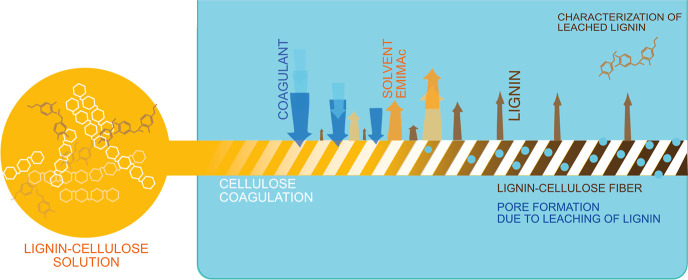

## Introduction

Manufacturing of carbon fibers from biobased raw materials offers a chance of lightweight composites with lower environmental impact compared to conventional and fossil-based carbon fiber.^1–3^ Man-made cellulose fibers may be used to manufacture carbon fibers; however, cellulose fibers suffer from a low carbonization yield.^4–7^ To overcome this issue, carbon fiber precursors have been created from cellulose blends in combination with other biopolymers, the most studied combination being lignin–cellulose.^8,9^ Using lignin is beneficial due to its high carbon content and since it is a byproduct from wood pulping and currently not widely used in any valuable product.^10,11^

Lignin and cellulose can be spun into continuous fibers after being dissolved together in, for example, an ionic liquid,^12–14^ or *N*-methylmorpholine *N*-oxide monohydrate.^15,16^ The fibers can be produced via wet spinning; however, in recent years, the focus has been on the air-gap spinning process since it gives the possibility of highly aligned molecular orientation in the fibers, which is favorable for carbon fibers.^17,18^ The fibers show promising characteristics as carbon fiber precursors in terms of enhanced spinning performance and high char yields due to synergies between lignin and cellulose.^19,20^ Some challenges with lignin–cellulose fibers have been identified and addressed; for example, spin finishes have successfully been applied to prevent adhesion of fibers.^21,22^ Furthermore, there is ongoing research on improving the mechanical properties of lignin–cellulose-based carbon fibers, and the tensile strength and modulus can likely be improved by optimizing the carbon fiber conversion.^14,23,24^

One of the remaining issues is leaching of lignin in the coagulation bath during fiber spinning. Previous studies have shown a tendency for high lignin loss for fibers with a lower lignin-to-cellulose ratio; also, hardwood lignin has been found to be more prone to leaching compared to softwood.^25,26^ The use of fractionation to render a higher-molecular-weight lignin is beneficial in terms of less lignin leaching.^12^ A higher molecular weight in combination with lower hydroxy content has previously also been found to limit lignin leaching during spinning.^27^ Furthermore, there are indications that the use of higher-molecular-weight lignin may produce carbon fibers with better mechanical properties.^28–30^ Although fractionation or modification of lignin would require an additional step in the lignin extraction process, the effort could thus be rewarded with lignins suitable for different applications.^31,32^

The aim of this work was to study how leaching of lignin varies depending on molecular weight and functional groups during air-gap spinning of cellulose–lignin precursors for biobased carbon fibers. Softwood kraft lignin was fractionated with solvent precipitation, and another lignin sample was isolated from wheat straw by using soda pulping. Complementary to previous studies in the field, a thorough analysis of the consequences of lignin leaching on the fiber structure is included, and the fiber morphology was analyzed in wet and dry states. Furthermore, the leaching of lignin was studied during fiber spinning and using a model setup with magnetic resonance imaging.

## Experimental Section

### Materials

Softwood kraft lignin (SKL) was obtained via the LignoBoost process from LignoDemo (Bäckhammar, Sweden). Detailed characterization of the lignin is available in a previous publication.^12^ Wheat straw was received from a farmer in Skåne, Sweden. A dissolving grade pulp (intrinsic viscosity of 465 mL/g according to ISO 5351:2010) from Georgia Pacific (Atlanta, Georgia, USA) was used as the cellulose source. Other materials are found in the Supporting Information.

### Wheat Straw Lignin Extraction and Precipitation

Wheat straw was treated following the procedure of Wojtasz et al.^33^ In short, the wheat straw was cut, submitted to dilute acid hydrolysis, and thereafter treated in an aqueous solution of NaOH (4 wt %). The alkaline cooking mixture was collected as a black liquor. The lignin from the black liquor was then precipitated by adding an aqueous solution of H_2_SO_4_ (2% vol %) until pH = 2 was reached. The mixture was then filtered and washed with neutral water. The final precipitated lignin was then dried in an oven at 105 °C for 5 h.

### Lignin Fractionation

Fractionation of SKL into four fractions was performed following the procedure by Cui et al.^34^ The ASKL Hex250 fraction corresponds to high-molecular-weight (HMW) SKL, and the ASKL PI corresponds to low-molecular-weight (LMW) SKL. The remaining lignin fractions were not used. A total of 80 g of nondried SKL (dry content of 61%, Table S1) as well as dried SKL (4 h 105 °C) was submitted to solvent fractional precipitation, and four fractions were obtained.

Information about the methods used for lignin characterization can be found in Supporting Information.

### In Situ Characterization of the Lignin Leaching during Coagulation

Magnetic resonance imaging (MRI) experiments were run on a Bruker Avance III 300 MHz, with a ^1^H transmit/receive probe with a 66 mm inner diameter. In situ MRI measurements were conducted on a model setup for fiber coagulation because the spatial resolution of the MRI limits direct measurement of the air-gap spinning process. The model setup was built based on a previous publication to study a cellulose-based system (see Figure 4 and Hedlund et al.^35^ for more details). Our setup consisted of a 50 mL vial functioning as the coagulation bath and a piston mounted to the vial’s lid. A cylinder, with a radius 1 mm larger than that of the piston, was filled with a solution of 1-ethyl-3-methylimidazolium acetate ([EMIM]­OAc), cellulose, and lignin. The piston was then pushed through the cylinder, forming a 1 mm thick film on the surface of the piston, which was then immersed in the vial (see Figure 4 for more details).

This setup was used for the coagulation of cellulose and lignin–cellulose films in two different solvents, water and ethanol. Ethanol was chosen since lignin is more soluble in ethanol compared to water, previously shown for the SKL.^36,37^

Two MRI protocols were used to characterize the mass transport in the coagulation bath. Voxel spectroscopic MRI was applied using the point-resolved spectroscopy sequence with water suppression. Several ^1^H spectra were recorded in a selected voxel of 3 mm^3^ in the coagulation bath during 30 min of coagulation (see Figure 4 for placement of the voxel). Coagulation of a cellulose solution was used as a reference. The [EMIM]­OAc peak areas for the cellulose–lignin films were normalized with the equilibrium [EMIM]­OAc peak areas for cellulose, accounting for the difference in [EMIM]­OAc concentration: 92 wt % for cellulose solution and 84 wt % for the cellulose–lignin solutions.

The second protocol is based on single-shot RARE (Rapid Acquisition with Relaxation Enhancement) images recorded with a RARE factor equal to the number of phase encoding steps to acquire a 2D image within one repetition time of 10 s, so-called one-shot. The RARE protocol was combined with saturation slices; i.e., the MRI signal of either water or ethanol is suppressed, resulting in regions with no signal. The saturation effect was initiated by multiple axial slices and was observed in the sagittal slice. RARE images were acquired repeatedly throughout the coagulation process for 20 min.

### Fiber Spinning

The lignin was used at a dry content of 70% and the cellulose dissolving pulp at 94%. Thereafter, equal amounts of lignin and cellulose (dry weight) were swelled in water before mixing with [EMIM]­OAc, to a total polymer concentration of 16 wt % for all samples containing lignin. Pure cellulose fibers were spun from a solution with 8 wt % cellulose dissolving pulp. Dissolution was performed in a low-pressure reactor at 95 °C for 1.5 h with stirring at 20 rpm at approximately 50 mbar to facilitate water evaporation. Dissolution was confirmed by light microscopy in bright field and under crossed polarizers, which showed no birefringent or undissolved particles. Stirring was stopped, the solution was degassed at 60 °C, 50 mbar for 30 min, and it was transferred to the spinning cylinder with limited introduction of air.

The spinning equipment included a piston pump, a coagulation bath, and a take-up roll. The solution was extruded at 45 °C at 4 m/min through a die consisting of 33 capillaries with diameters of 120 μm with L/D 2 and collected at 16 m/min, i.e., a draw ratio of 4. The solution passed through a 1 cm air gap before coagulation in deionized water at temperatures of 1–5 °C. Fibers were washed in deionized water at room temperature. Fibers for wet analysis were kept in deionized water at 8 °C before analysis. Drying of the fibers was performed at 60 °C for 30 min.

Information about methods for the analysis of fiber properties and morphology analysis can be found in Supporting Information.

## Results and Discussion

In this work, we have fractionated commercial softwood Kraft lignin (SKL) into a high- and low-molecular-weight fraction, denoted HMW and LMW, respectively, and extracted lignin with the soda pulping process from wheat straw, denoted WSL. These lignins together with cellulose were spun into fibers—precursors for biobased carbon fibers. We identified two potential factors determining the fiber morphology: (i) lignin’s properties and (ii) mass transport during fiber spinning and coagulation. First, the lignin yield and fiber morphologies were analyzed and then linked with the results of the lignin properties and the mass transport.

### Amount of Lignin Retained in Spun Fibers

Details about the solvent fractionation of the softwood Kraft lignin are described in the Supporting Information. The chemical composition of the lignins in terms of acid-insoluble lignin (AIL) and acid-soluble lignin (ASL) is included in Table 1 and corresponds well to previous findings.^12^ The LMW contains the highest amount of ASL, as expected.^38^ The low total amount of AIL and ASL in the WSL indicates a high ash content.

**Table 1:** Acid-Insoluble Lignin (AIL) and Acid-Soluble Lignin (ASL) of the Lignins and Lignin in the Spun Fibers Including the Total Lignin Content in the Fibers and the Yield^a^

sample	AIL (wt %)^a^	ASL (wt %)^b^	theoretical AIL content (wt %)	AIL yield (%)
SKL	87.9	6.0	n.a.	n.a.
HMW	93.2	1.9	n.a.	n.a.
LMW	76.5	11.4	n.a.	n.a.
WSL	68.0	8.1	n.a.	n.a.
SKL fiber	46.2	0.11	45	100
HMW fiber	46.3	0.09	47	98.5
LMW fiber	35.4	0.05	38	93.2
WSL fiber	15.0	0.13	34	44.1

a Standard deviation of methods from duplicates: 1.1% and 0.1%.

Attempts for quantification of lignin retained in the fibers were performed using acid hydrolysis on lignin and fibers and UV-absorption on the coagulation bath. Already during spinning, a clear difference was seen between the SKL and HMW fibers and the LMW and WSL fibers, where spinning of the latter two types rendered a colored coagulation bath. The AIL content of the fibers, together with the AIL yield, is presented in Table 1.

The AIL yield in the fibers follows the same trend—HMW, SKL, LMW, and WSL—as the lignin yield based on the UV analysis of the coagulation bath, summarized in Table S5. The lowest AIL yield was observed for the WSL fiber, albeit having the largest molecular weight. This observation indicates that molecular weight might not be the decisive factor, which is further elucidated in the following sections. HMW fiber leached less lignin compared to the SKL fiber based on the UV-analysis. This is likely due to the higher ASL content of the SKL fiber compared to the HMW fiber, shown in Table 1. The ASL content in the fibers is very low compared to the lignins used, indicating a large loss of this type of lignin during spinning. Unfortunately, from the AIL and ASL content alone, the difference in lignin leaching for the SKL and HMW fiber cannot be distinguished, stressing the limitations of these methods.

### Properties and Morphology of Spun Fibers

The mechanical properties of the fibers are listed in Table 2, with full stress–strain curves available in Figure S1. The diameter, tenacity, and modulus were converted to SI units using a density of 1.5 g/cm^3^ and assuming a circular cross section (Figure S2). The linear density of the fibers, i.e., the titer, is larger for fibers retaining more lignin, with the WSL fiber showing the lowest titer in accordance with the AIL yield in Table 1. The elongation appears to be similar for all fibers. The tenacity and modulus are highly influenced by the lignin content due to cellulose having a higher molecular weight, exhibiting molecular orientation within the fiber, and being semicrystalline.^13,25,39,40^ Thus, fibers containing less lignin are expected to have higher tenacity and modulus, in line with our results.

**Table 2:** Mechanical Properties of Spun Fibers, 10 Fibers Tested for Each Sample^a^

	titer		elongation		tenacity		tensile modulus		
sample	dtex *μm*	±	%	±	cN/tex *MPa*	±	cN/tex *GPa*	±	normalized tenacity cN/tex
SKL fiber	5.1	0.7	5.7	1.4	20.8	2.0	810	52	38.7
	*20.8*	*1.3*			*312*	*29*	*12.2*	*0.8*	

HMW fiber	5.7	1.7	5.8	2.0	16.7	2.8	716	143	31.1
	*21.5*	*2.4*			*285*	*67*	*10.1*	*1.7*	

LMW fiber	4.7	1.0	7.5	3.1	18.1	3.0	778	70	28.0
	*19.8*	*2.1*			*272*	*44*	*11.7*	*1.1*	

WSL fiber	4.5	0.9	6.1	1.7	23.1	3.7	960	135	27.2
	*19.2*	*2.0*			*352*	*56*	*14.7*	*2.1*	

cellulose fiber	3.0	0.2	6.2	0.4	32.6	2.3	1324	90	32.6
	*16.0*	*0.4*			*489*	*34*	*19.9*	*1.4*	

a Tenacity was normalized to the cellulose content, considering the acid-insoluble lignin content summarized in Table 1.

If lignin’s contribution to fiber tenacity is negligible, the tenacity may be normalized to the cellulose content in the fibers using the AIL content in Table 1. The normalized tenacity of the SKL and HMW fibers is similar to the tenacity of the pure cellulose fiber, indicating that the cellulose phase in the fiber determines its strength. The WSL fiber is the weakest among the lignin–cellulose fibers, followed by the LMW fiber. Thus, lignin leaching seems to have a negative impact on the mechanical properties of the fibers.

Apart from the mechanical properties, the fiber morphology was analyzed in the wet and dry states. All fibers exhibited a predominantly circular shape with no visible surface pores (Figure S2). Using light microscopy on the wet fiber, it was possible to see a lot of inhomogeneities in the WSL fibers, possibly pores (Figure 1 left), not shown in the other lignin–cellulose fibers (Figure S3a).

**Figure 1:**
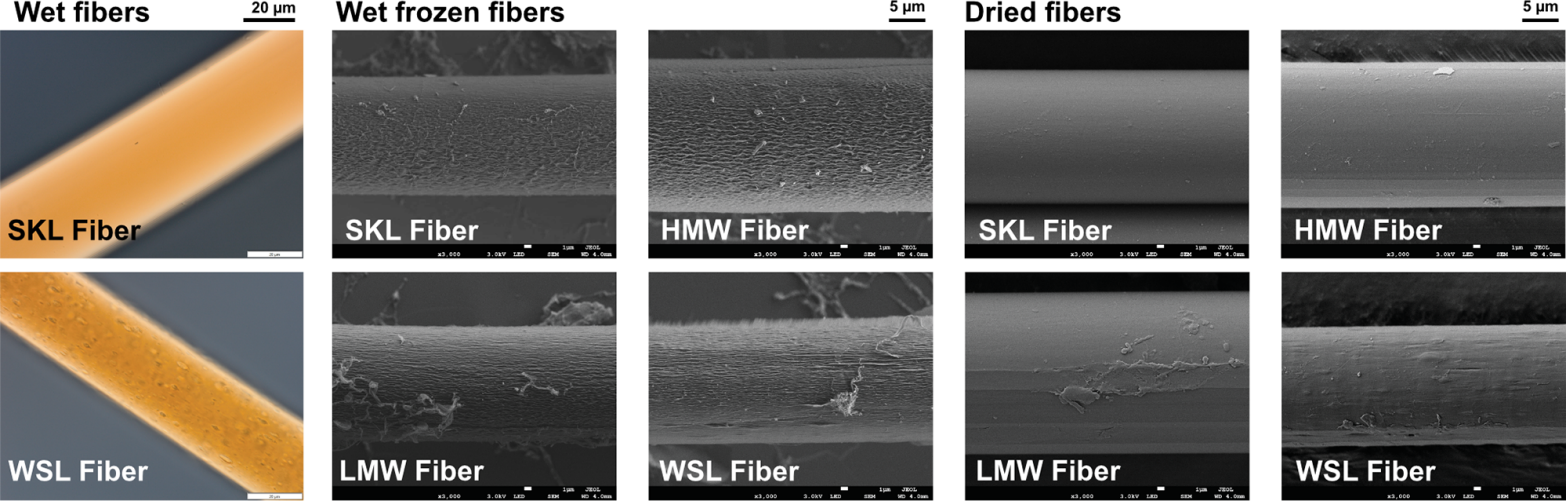
Light microscopy of wet fibers (left), SEM imaging of a metal replica made on frozen never-dried wet fibers (middle), and dried fibers (right).

To disclose the fiber surface, metal replicas of the fibers were made for never-dried fibers, which were frozen and observed in SEM. The observed structure of the surface of the frozen fibers may be a result of the sample preparation technique; however, the large difference between the different samples likely arises from the properties of the sample.^41^ The never-dried lignin–cellulose fiber replicas revealed loose fibrillar structures, particularly the WSL fiber, which might be related to the pilling of cellulose fibrils (Figure 1 middle). Likely, lignin leaching causes a more open fibrillar network of cellulose, prone to pilling and abrasion. All lignin–cellulose fibers also exhibited an increased surface roughness in comparison to that of the cellulose fibers (Figure S3b), which had a more fibrillar surface.

The surface of dried fibers spun with SKL and HMW lignin had a smooth surface (Figure 1, right). Some unevenness was detected for the LMW fibers, while the WSL fibers exhibited a very uneven fiber surface in the dried state.

The fiber analysis suggests that large amounts of lignin leaching result in fiber defects, as was seen for the WSL fiber. Presumably, even minor lignin leaching during the spinning process could induce small-scale structural irregularities locally within the fiber, having a measurable impact on fiber morphology. Since the tenacity of carbon fibers is known to be very sensitive toward defects and pores,^42,43^ leaching of lignin should thus be limited.

What causes these defects—is it only related to the amount of lignin leaching, or do the dynamics during the coagulation play a role as well? To shed light on these questions, we further analyzed the lignin fractions and the lignin retained in the fibers, and the mass transport during coagulation.

### Lignin Characterization

The lignins used and lignin retained in the fibers were further characterized using gel permeation chromatography, 2D NMR, and thermogravimetric analysis (TGA), to understand why some lignins leach more than others. Gel permeation chromatography was used to assess the molecular weight distribution of WSL, SKL, and SKL fractions and lignin in fibers; see Figure 2. The data clearly show that the fractionation of SKL afforded two distinct fractions with a lower polydispersity index than the original unfractionated SKL, Table 3.

**Figure 2:**
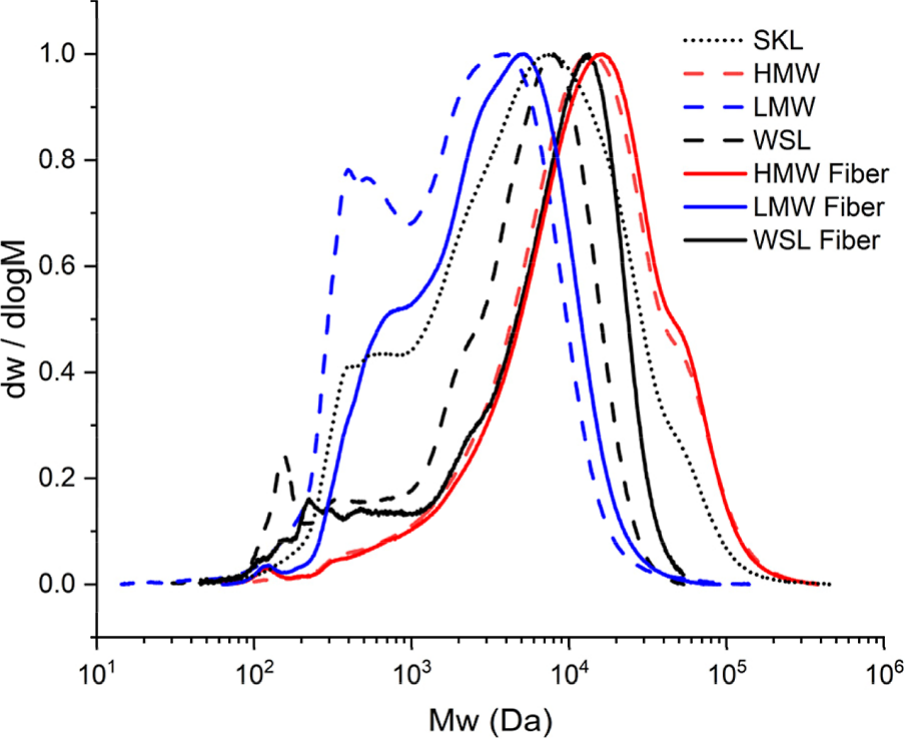
Normalized molecular weight distribution of lignin samples (dotted and dashed lines) and lignin in spun fibers (solid lines).

**Table 3:** Molecular Weight Averages of Lignin Samples (kDa), *M*_p_ (Peak Molecular Weight), *M*_n_ (Number-Average Molecular Weight), *M*_w_ (Weight-Average Molecular Weight), *M*_*z*_ (*Z*-Average Molecular Weight), and PDI (Polydispersity Index)

sample	*M*_p_	*M*_n_	*M*_w_	*M*_*z*_	PDI (*M*_w_/*M*_n_)
SKL	7.6	1.6	10.5	34.0	6.4
HMW	13.3	4.2	19.0	47.4	4.5
LMW	3.5	1.0	3.4	8.9	3.5
WSL	7.9	1.2	6.5	11.7	5.5

The LMW fraction has a lower polydispersity index (PDI = *M*_w_/*M*_n_) than the HMW fraction, in agreement with the results by Cui et al.^34^ The PDI is influenced by the insolubility of larger lignin molecules with increasing hexane concentration during the fractionation, resulting in a larger decrease of *M*_w_ (weight-average molecular weight) than the decrease in *M*_n_ (number-average molecular weight). Despite similar results, the obtained PDI values in this work are higher than the ones for similar fractions reported by Cui et al.^34^ (1.8 and 1.3 for HMW and LMW, respectively). Notably, WSL has a more homogeneous molecular weight distribution and higher molecular weight compared to SKL, apart from a distinct small share of low-molecular-weight lignin.

The lignin remaining in the fibers was also analyzed in terms of molecular weight and presented in Figure 2. Also, here, a clear difference between HMW fiber and LMW/WSL fiber was found. While the lignin in the HMW fiber showed a similar distribution to the original HMW lignin, the molecular weight of the lignin in the LMW fiber and WSL fiber has shifted toward higher molecular weight. The LMW fiber showed a clear loss of low-molecular-weight fractions, and previous studies have drawn the conclusion that lignin leaching is caused mainly by the low molecular weight.^37^ However, it is also known that the low-molecular-weight fraction of kraft lignin is enriched in polar functional groups.^44^ Furthermore, the WSL fiber showed more of an overall shift of the molecular weight distribution, less dependent on the molecular weight, pointing toward broad overall leaching, likely indicating a contribution of the different chemical groups in the lignin samples.

To evaluate the differences in chemical composition, the content of different hydroxy groups was assessed by ^31^P NMR for the four lignin samples (SKL, HMW, LMW, and WSL) and is summarized in Table 4 and Figure S4. For LMW lignin, a higher content of carboxylic acid groups was observed compared to HMW lignin, in line with previous studies.^34,44,45^ LMW lignin also has a higher content of phenolic hydroxy groups than the HMW fraction.^46^ Both groups contribute to a higher solubility in polar protic solvents, which is also further favored by their lower molecular weight.

**Table 4:** Content of Aliphatic, Phenolic Hydroxy, and Carboxylic Acid Groups of Lignin Samples

sample	aliphatic –OH	phenolic –OH	–COOH
SKL	1.86	4.29	0.43
HMW	2.21	3.76	0.39
LMW	1.23	4.92	0.66
WSL	0.92	1.49	1.49

While WSL has the lowest content of phenolic hydroxy groups, it has more than twice the carboxylic acid content, as expected for soda lignin.^47^ The higher content of carboxylic acid groups likely makes the WSL more soluble in the coagulation bath and the lower-molecular-weight fractions, thus promoting more extensive lignin leaching.

^1^H NMR spectra of the lignin samples are not sufficient to provide detailed information on their different moieties (Figure S5), and further characterization of the lignin samples was performed using 2D NMR. The ^1^H–^13^C HSQC spectrum shows that the aromatic groups in the SKL sample are mainly composed of G-units and some terminal aldehydes (benzaldehyde and cinnamyl aldehyde) and acetovanillone (Figure 3a, and for more details, see Figure S6). Stilbene and aryl enol ether units were also found.

**Figure 3:**
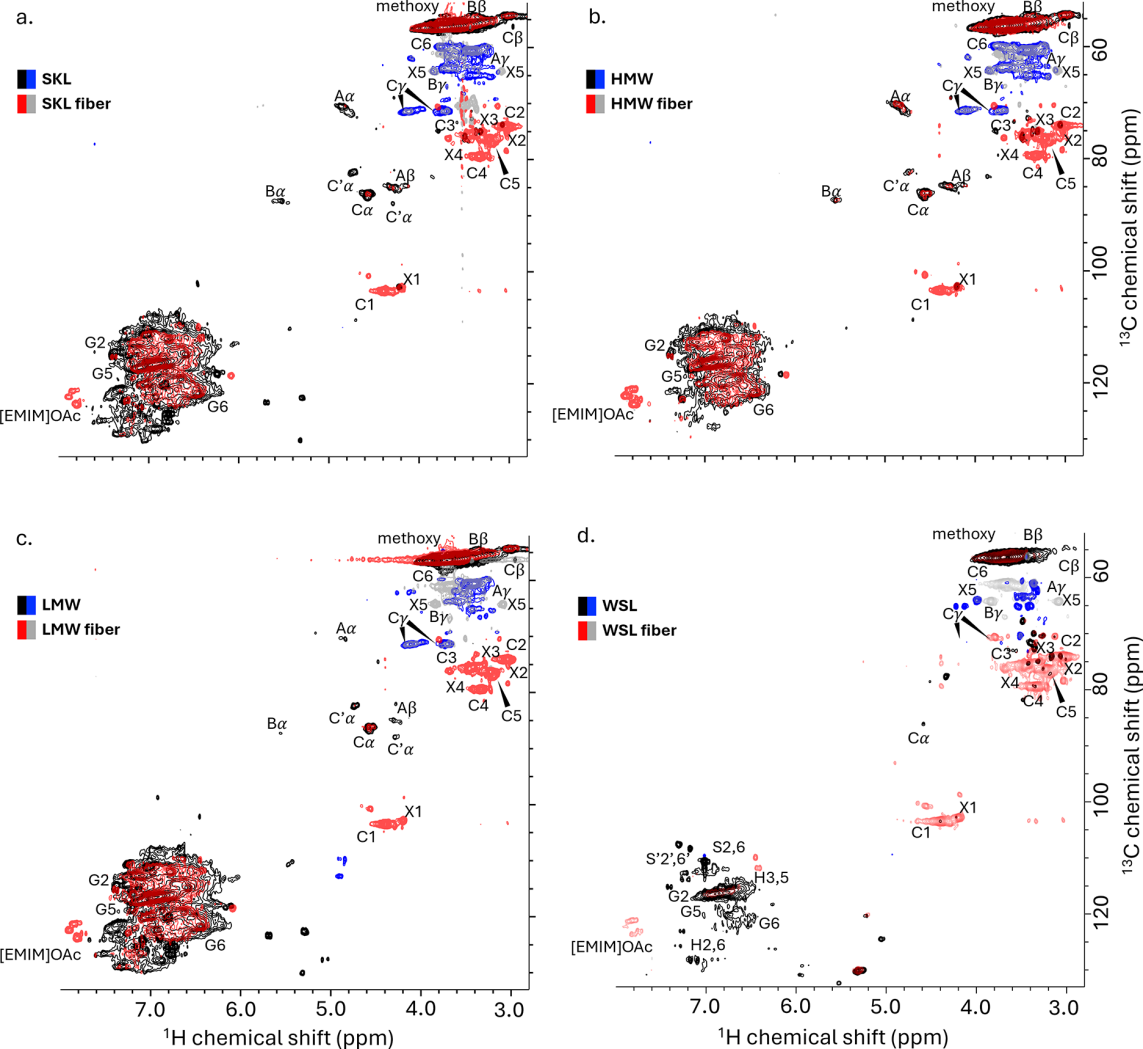
2D ^1^H–^13^C HSQC spectra of spun fibers (red–gray) overlaid with the HSQC spectra of the corresponding lignins (black-blue) dissolved in [P_4444_]­[OAc] diluted with DMSO-*d*_6_. (a) SKL lignin and fiber, (b) HMW lignin and fiber, (c) LMW lignin and fiber, and (d) WSL lignin and fiber. C refers to cellulose, X to xylan, and Aα,β,γ, Bα,β,γ, and Cα,β,γ correspond to some lignin linkages. G refers to the G-units, S to the S-units, and H to the H-units present in lignin. The numbers refer to the atomic number in the molecular structure. Blue and gray are CH_2_ groups, while black and red are CH or CH_3_ groups.

The SKL fractions, LMW and HMW, have similar compositions to the SKL sample, although with some differences (Figure 3a–c and for more details, see Figures S6–S8). The HMW sample lacks the presence of the cinnamyl aldehyde group, and it has weaker signals of the stilbene and benzaldehyde groups. All three samples exhibit the same type of linkages (β-*O*-4, β–β′, and β-5), yet the LMW sample has fewer amounts of those. This was also observed earlier by Crestini et al.,^46^ which could indicate a more branched structure for the LMW compared to HMW.

For WSL, as expected due to different origins, the aromatic region of the HSQC is more diverse with both H-, G-, and S-units and the terminal groups *p*-coumarate and ferrulate (Figures 3d and for more details, see S9). The linkages are mainly condensed β–β′ and β-*O*-4 type, although the peaks for the α and β hydrogens from the β-*O*-4 linkage were absent. None of the lignin samples had dibenzodioxocin linkages, being advantageous for carbon fiber precursors because less branched chains can reduce defects and improve the mechanical properties of the fibers.^48^ The HMBC spectra for the four lignin samples were used to confirm the linkages between the different units (Figure S10).

Lignin is a complex mixture with a molecular weight distribution and multiple functional groups. To determine if fractions with specific functional groups have leached, the spun fibers were dissolved as reported previously by Fliri et al.^49^ This protocol was also used to investigate thermostabilized cellulose fibers^49^ and cellulose–lignin blends in thin films.^50^

The ^1^H–^13^C HSQC spectra of the dissolved spun fibers (red–gray) were overlaid with the corresponding lignins (black–blue) as shown in Figure 3. For the fibers, peaks arising from cellulose and xylan are visible. For SKL fiber and LMW fiber, comparing lignin peaks, aldehydes, stilbenes, and acetovanillone have leached into the coagulation bath, and if remaining, the concentration is below the detection limit. The HSQCs for HMW and HMW fiber appeared very similar, which agrees with the low leaching and high amount of lignin retained in the fiber.

The highest degree of lignin leaching was, in line with previous results, observed for the WSL fibers, which exhibited a significant loss of characteristic lignin peaks and several unassigned peaks. In addition, we performed solid-state ^13^C CP NMR on the spun fibers, and the spectra showed both lignin and cellulose being present. Unfortunately, ^13^C CP NMR is not quantitative, and due to the complex nature of the samples, the observed peaks are broad and featureless (see Figure S11). Nevertheless, inspecting the peak shape of C4, all fibers appeared to have a similar cellulose crystallinity.

The temperature for the onset of degradation, *T*_onset_, and char yield of the different lignins are summarized in Table 5, and the full TGA spectra are available in Figure S12. The WSL contained large amounts of residue after the oxygen step in the TGA, indicating a high ash content. The residual mass was not accounted for in the char yield because the ash content should have leached out during spinning. The WSL exhibited the lowest *T*_onset_, which has been observed in previous studies, likely from a combination of the high amounts of carboxylic acid groups forming CO_2_ during degradation^51,52^ and the fraction of low-molecular-weight lignin.^53^

**Table 5:** Thermal Characterization and Chemical Composition of the Lignins and the Spun Fibers^b^

sample	*T*_onset_ (°C)	*Y*_char_ (%)^a^	theoretical *Y*_char_ (%)	*T*_g_ (°C)
SKL	269	43.2	-	163
HMW	229	42.8	-	157
LMW	247	37.3	-	105
WSL	188	37.0	-	132
SKL fiber	263	35.9	33.9	-
HMW fiber	269	32.1	33.7	-
LMW fiber	277	31.8	29.9	-
WSL fiber	262	27.6	27.6	-
cellulose fiber	285	25.9	-	-

a At 700 °C.

b The theoretical char yield was calculated using the char yields of the respective lignins and the cellulose fiber, using the AIL content of the fibers.

Furthermore, upon comparison of the fibers to a pure cellulose fiber, the *T*_onset_ of degradation decreased. However, for the WSL fiber compared to WSL, the *T*_onset_ increased, likely due to its low lignin content.

The char yield increased for fibers containing lignin compared with a pure cellulose fiber. The SKL has the highest char yield, and the SKL fibers have the highest char yield of all the lignin–cellulose fibers, indicating the most retained lignin in the fiber. The mass loss peak also shifted to higher temperatures for the lignin–cellulose fibers compared to the pure cellulose fiber deduced from the derivative thermogravimetric curve shown in Figure S13. This shift has been attributed to interactions between lignin and cellulose,^54^ and as can be seen in Table 5, the char yield is higher than the expected theoretical char yield calculated for the SKL and LMW fiber.

The *T*_g_ of the different lignins, calculated from the DSC curves (Figure S14), summarized in Table 5, are correlated to molecular weight.^38^ However, HMW has a similar *T*_g_ as SKL despite being of higher molecular weight, revealing a difference in their chemical structure. A similar *T*_g_ indicates a higher flexibility of the lignin chains in the HMW compared to SKL. HMW does have a higher content of aliphatic hydroxy groups compared to SKL, which could be the result of the less condensed structure (i.e., lower amount of β–β′ and β-5 linkages and stilbene structures).^45^ A higher *T*_g_ is beneficial in terms of carbon fiber manufacturing since it allows faster stabilization without fiber fusion;^30^ however, the current type of lignin–cellulose fibers has been reported to be successfully carbonized without stabilization.^55^

When summarizing the analysis of the lignin in fibers, it is difficult to distinguish between the SKL and HMW in terms of the amount of lignin leaching, with possibly slightly more SKL retained in the fibers, comparing AIL content and char yield. Thus, no clear benefit of the lignin fractionation on the lignin yield was found, despite the higher molecular weight of HMW compared to SKL. Furthermore, the WSL leaching was more pronounced than that of LMW despite WSL having a higher average molecular weight compared to LMW. The results consequently underline that lignin leaching is primarily caused by the polar functional groups, such as carboxyl groups and aldehydes, and seems to promote lignin leaching of a broad molecular weight range. Consequently, if a fractionation would render a high-molecular-weight lignin with a substantially lower amount of polar functional groups and a high amount of condensed moieties, a clearer benefit may be seen.

### Leaching of Lignin during Coagulation in a Model System

To gain a deeper understanding of the mass transport of the solvents and lignin leaching during spinning, the coagulation process was analyzed using MRI. The spatial resolution of the MRI limits observing fibers spun using air-gap spinning, and a model setup producing thin films instead was employed, as shown in Figure 4. Two MRI protocols were used to (i) monitor the relative [EMIM]­OAc concentration leaching during the coagulation process and (ii) the duration of the mass transport caused by solvent mixing and lignin leaching. For the first, we used voxel imaging to record a 1D ^1^H spectrum in a voxel. The voxel, here with a size of 3 mm^3^, was placed close below the piston where most of the mixing and solvent transfer occurs (for the placement of the voxel, see Figure 4, middle panel).

**Figure 4:**
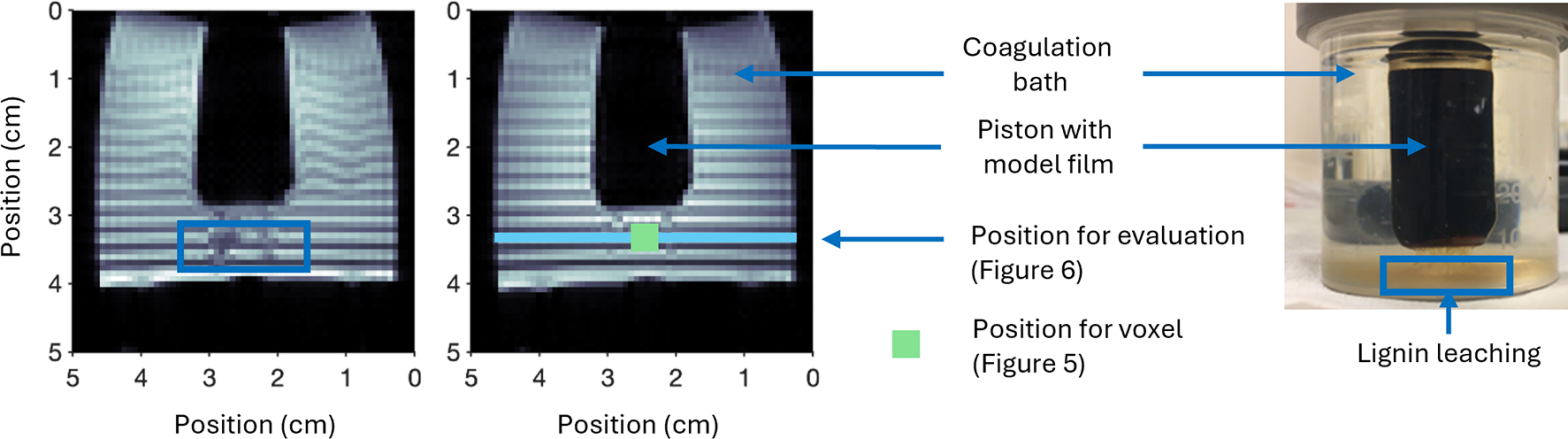
(Left) Two sagittal MRI images with saturation slices (dark stripes) at two different points in time using the model setup shown to the right to monitor the mass transport during coagulation of cellulose–lignin films. The position of the voxel tracking the [EMIM]­OAc relative concentration with time, shown in Figure 5, and the position for the evaluation of the mass transport with time, shown in Figure 6, are highlighted.

The spectra showed that the intensity of the [EMIM]­OAc peaks was continuously increasing with time until equilibrium was reached (see Figure S15). Unfortunately, lignin could not be observed in these ^1^H NMR spectra, most likely due to the low concentration and distribution in molecular weight. However, its presence was evident from the brown color in the coagulation baths (Figure 4) and further confirmed by both UV absorbance (Table S5) and high-field NMR spectroscopy measurements on the coagulation baths (Figure S16).

The integrals of the [EMIM]­OAc peaks represent the relative [EMIM]­OAc concentration with time using a pure cellulose solution as reference, shown in Figure 5. Already at the beginning of coagulation, the relative [EMIM]­OAc concentration in the coagulation bath when coagulating cellulose was higher compared to cellulose solutions containing HMW, LMW, or WSL. This suggests that [EMIM]­OAc was transported faster from the cellulose solution into the bath, which could be attributed to a higher [EMIM]­OAc concentration in the cellulose solution. After 30 min, the relative [EMIM]­OAc concentration in the coagulation bath for the HMW-cellulose solution stabilized, forming a plateau in contrast to the LMW- and WSL-cellulose solutions, continuing to increase likely due to intense lignin leaching. Additionally, the relative [EMIM]­OAc concentration was always higher for LMW- and WSL-cellulose solutions compared to HMW. These findings imply that intense lignin leaching correlates with faster transport of [EMIM]­OAc into the bath, which in turn also aligns with the defects on the fibers, facilitating enhanced [EMIM]­OAc mass transport.

**Figure 5:**
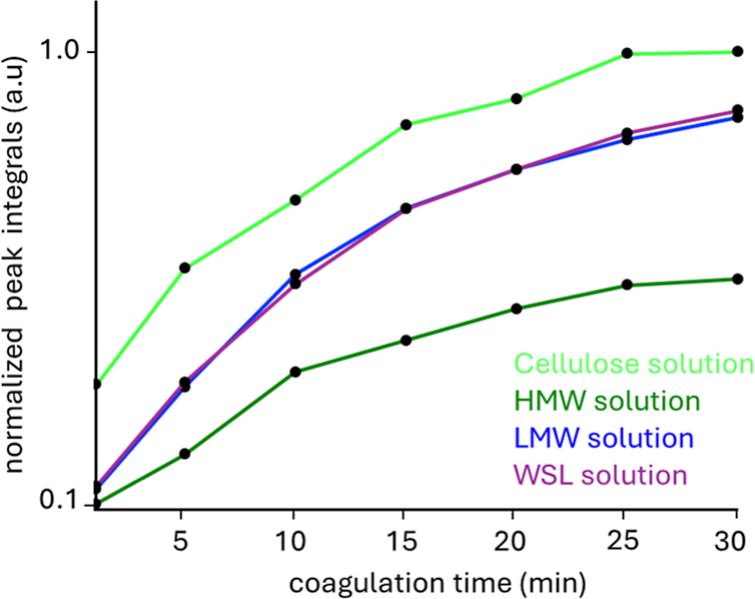
Evolution of the [EMIM]­OAc peak integrals obtained from ^1^H NMR spectra recorded in the voxel shown in Figure 4.

Results from our study so far suggest that the solubility of lignin might impact the leaching. Hence, we chose another coagulant, ethanol, in which lignin is more soluble compared to water, as shown earlier, for the SKL.^36,37^

The second MRI protocol—single-shot RARE with saturation slices—allows one to observe the duration of the mass transport. Typical RARE images are shown in Figure 4, left panel. Straight dark stripes entail no mass transport. We chose to follow the mass transport again close to the piston, and the MRI signal intensity throughout the dimension of the vial at this vertical position was plotted with time (see Figures 4 for more details and 6 for results). Regions highlighted in red correspond to high signal variation, indicating areas with intense mass transport seen for WSL- and LMW-cellulose solutions independently of the coagulant. In contrast, the signal varied less for the HMW-cellulose solution, suggesting a less intense mass transport. Unfortunately, no clear trend was observed for the two solvents.

**Figure 6:**
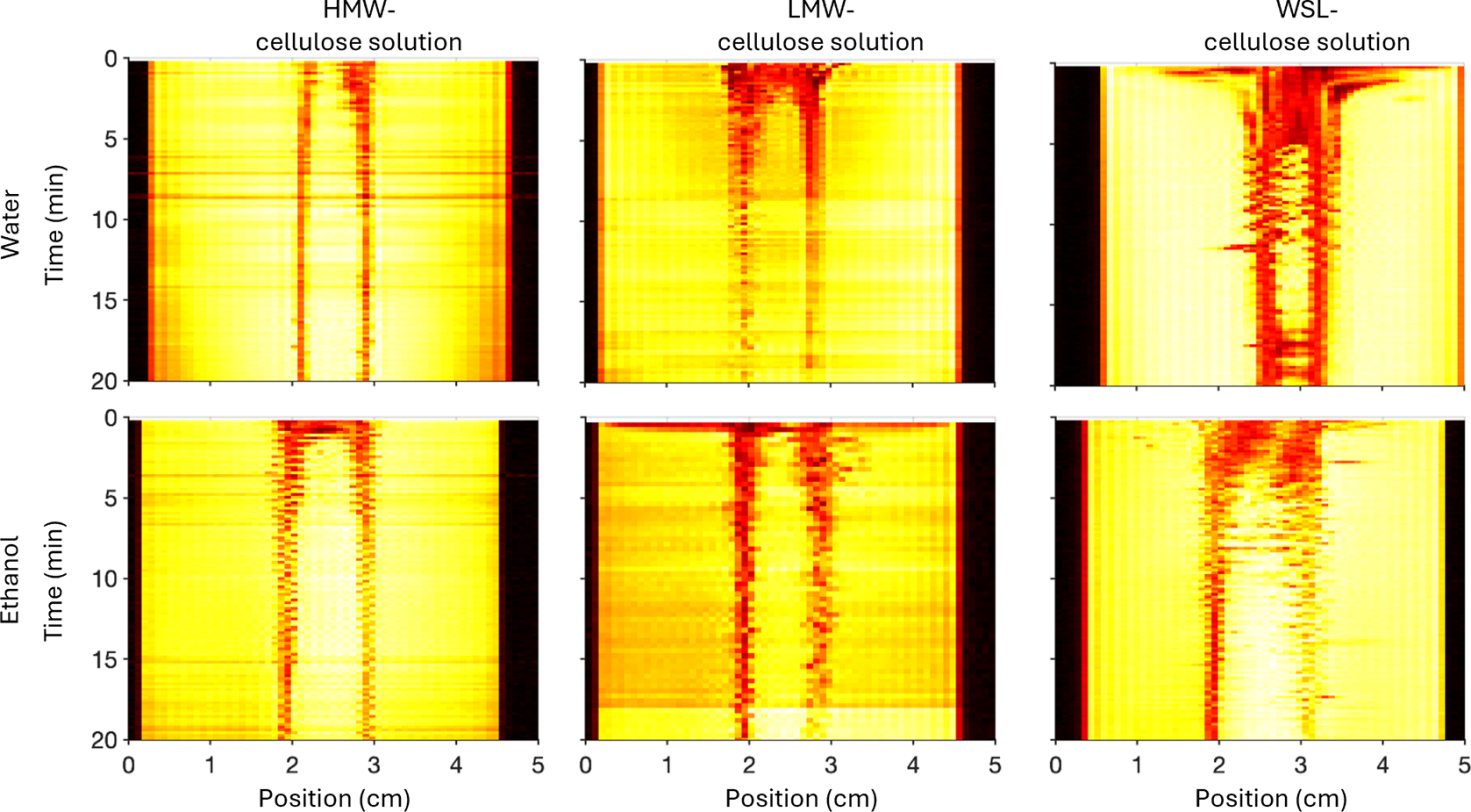
Evaluation of the mass transport by following one vertical position in the MRI images (see Figure 4) below the piston with time for water and ethanol for cellulose solutions with the same concentration of lignin and cellulose as HMW fiber, LMW fiber, and WSL fiber. Yellow corresponds to low mass transport and red to intense mass transport.

The overall observations and analysis from both MRI methodologies applied to the model setup align well with findings from the spun fibers, where the greatest lignin loss was observed for the LMW and WSL fibers. Assuming that a rigid cellulose network was formed rather quickly for all samples,^56^ continuous intense mass transport suggests ongoing lignin leaching leading to the formation of pores and defects in the fibers found for the WSL fiber.

## Conclusions

In this study, the cause of lignin leaching during air-gap spinning was elucidated by varying the molecular weight and the functional groups of lignin. Thorough analysis of lignin’s properties, lignin yield in the fibers, fiber morphology, and mass transport during solution coagulation concluded that lignin leaching depends strongly on the amounts of functional groups.

WSL leaching was more pronounced than LMW despite having a higher average molecular weight compared with LMW. Hence, the presence of carboxylic acid groups appears to strongly promote leaching across a wider molecular weight range. This suggests that not only the size but also the polarity of the lignin determine the lignin yield.

Furthermore, for lignins with a lower amount of polar functional groups, the lignin yield did not increase using a solely HMW fraction compared with the SKL. Instead, our results indicate that the number of condensed structures is also crucial. Hence, the choice of lignin, which should be considered in future work, consists of low amounts of polar functional groups and a higher amount of condensed lignin moieties, and it is beneficial to have a high molecular weight.

Leaching of lignin caused defects in the spun fiber. Lower final lignin content in spun fiber gave an apparent increase in tensile strength; however, when tenacities were normalized with cellulose content, fibers with the most substantial lignin leaching were weaker. For the WSL fibers, for which extensive lignin leaching occurred, defibrillation and pores in the spun fiber were observed in the wet and dry states. The mass transport analyzed with MRI suggests that the leaching of lignin occurs from a preformed cellulose network, thus introducing pores in the fiber. Therefore, limiting lignin leaching is essential not only to improve solvent recovery but also to produce defect-free precursors for high-quality carbon fibers.

## Supplementary Material



## Notes

Details on chemicals and solvents, compositional analysis, lignin fractionation yield data, molecular weight (GPC) methodology, chemical structure analysis (solution and solid-state NMR), acid-insoluble/soluble lignin content, fiber mechanical properties, fiber morphology analysis specifications (LM, SEM, replicas, cross sections), lignin leaching during spinning (UV/vis, conductivity), and coagulation bath analysis (NMR, MRI) (PDF)

## Abbreviations

AIL: acid-in-soluble lignin
ASL: acid-soluble lignin
HMW: high molecular weight
LMW: low molecular weight
SKL: softwood kraft lignin
WSL: wheat straw lignin
