# Supplementary material for: Impact of Lignin Type on Yield and Fiber Morphology in Biobased Carbon Fiber Precursors
Source: ACS Omega. 2026 Mar 25;11(13):20636–45. doi: 10.1021/acsomega.5c12442 (PMC13063169; doi:10.1021/acsomega.5c12442)
Supplement: Supplementary file 1 [file ao5c12442_si_001.pdf]

Supporting Information

Impact of lignin type on yield and fiber morphology in biobased carbon fiber precursors

Jenny Bengtsson[a,b], Leandro Cid Gomes[a], Feryal Guerroudj[a], Hanna Ulmefors[c,b], Annika I. Altskär[d], Michael Hummel[e], Diana Bernin[a]*

Jenny Bengtsson: a. Department of Chemistry and Chemical Engineering, Chalmers University of Technology, 412 96 Gothenburg, Sweden.
b. RISE Research Institutes of Sweden, Department of Polymer, Fiber and Composites, 431 53 Mölndal, Sweden.

Leandro Cid Gomes: a. Department of Chemistry and Chemical Engineering, Chalmers University of Technology, 412 96 Gothenburg, Sweden

Feryal Guerroudj: a. Department of Chemistry and Chemical Engineering, Chalmers University of Technology, 412 96 Gothenburg, Sweden

Hanna Ulmefors: c. Chalmers Industriteknik, 412 58 Gothenburg, Sweden.
b. RISE Research Institutes of Sweden, Department of Polymer, Fiber and Composites, 431 53 Mölndal, Sweden.

Annika Altskär: d. RISE, Research Institutes of Sweden, Department Agriculture and Food, 412 76 Gothenburg, Sweden

Michael Hummel: e. Department of Bioproducts and Biosystems, Aalto University, P.O. Box 16300, 0076 Aalto, Finland.

Diana Bernin: a. Chalmers University of Technology, Department of Chemistry and Chemical Engineering, 412 96 Gothenburg, Sweden

*Corresponding author: jenb@chalmers.se

KEYWORDS: Lignin, cellulose, fiber spinning, lignin leaching, coagulation, NMR

Materials

Acetone (USP ACS, VWR, Sweden) and hexane (98.5%, Fisher, Sweden) were used as solvents for the fractionation of SKL. Sodium hydroxide (≥ 98%, Merck, Sweden) and sulfuric acid (95–97%, Merck, Sweden) were used in the extraction and precipitation of lignin from wheat straw (WSL). Chloroform-d (99.8 atom % D), dimethyl sulfoxide-d6 (99.5 atom % D), pyridine (anhydrous, 99.8%), 2-chloro-4,4,5,5-tetramethyl-1,3,2-dioxaphospholane (95%), chromium(III) acetylacetonate (99.99%) and cholesterol (≥ 99%) were purchased from Meck and used for NMR analysis. The solvent used for fiber spinning was 1-ethyl-3-methylimidazolium acetate ([EMIM]OAc, ≥98%, Proionic, Raaba-Grambach, Austria). All chemicals were used as received.

Table S1. Water content in SKL

Sample	m_0 (g)	m_{lost} (g)	m_{final} (g)	Water (wt%)	T	t (h)
SKL	3.5279	1.3851	2.1428	39	105 °C	5
SKL	3.9994	1.5103	2.4891	38	50 °C	~24

Solvent fractionation of lignin

The HMW and LMW fractions accounted for 82 wt% of the total fractions recovered and 58 wt% of the total dry content used in the fractionation. The overall fractionation yield was 71 wt% (Table S2). Another fractionation was performed with dried SKL with a similar overall yield of 80 wt% (Table S3), with a higher amount of acetone insoluble lignin fraction (AIKL), which indicates that drying at 105 °C led to condensation of low molecular weight fractions. Both fractionation yields are lower than the ones reported by Cui *et al.*[1] (up to 95 wt%), likely indicating a higher amount of volatile content in the SKL [1]. Fractions AIKL and ASKL PII (see Table S2) were not used in this work. The non-dried SKL and the LMW and HMW fractions thereof were used for fiber spinning.

Table S2. Fractionation of non-dried SKL

Lignin type	m (g)	Yield (wt%)	Dry mass (g)	Yield dry (wt%)	Recovered distribution
SKL	80.0		48.8		
AIKL	0.6	0.75	0.6	1.2	1.7%
ASKL Hex250 (HMW)	17.0	21.2	17.0	34.8	50%
ASKL P I (LMW)	10.8	13.5	10.8	22.1	31.8%
ASKL P II	5.6	7.0	5.6	11.5	16.5%
**Total recovered**	**34**	**42.4**	**34**	**69.7**	**100%**

Table S3. Fractionation of dried SKL at 105 °C

Lignin type	m (g)	Yield (wt%)
SKL	4.63	
AIKL	2.22	48.0

ASKL Hex250 (HMW)	0.13	2.7
ASKL P I (LMW)	0.41	8.9
ASKL P II	0.57	12.4
ASKL P III	0.35	7.6
**Total recovered**	**3.69**	**79.7**

Lignin characterization

Lignin molecular weight

Gel permeation chromatography (PL-HPC 50 Plus Integrated GPC system, Polymer Laboratories, Varian Inc.) was used to assess the molecular weight distribution of the lignin samples. The system is equipped with two 300 × 7.5 mm PolarGel-M columns, one 50 × 7.5 mm PolarGel-M guard column, one refractive index (RI) detector and one UV detector operating at 280 nm. Dimethyl sulfoxide with 10 mM LiBr was used as the mobile phase and the flow rate was 0.5 mL min-1 at 50 °C. The UV detector was calibrated based on the calibration of the RI detector with 10 Pullulan standards ranging from 0.180 to 708 kDa (Varian PL2090-0100, Varian Inc). Samples were prepared by dissolving ca. 10 mg of the lignin sample (sample weights are available in Table S4) in 1 mL of the mobile phase under stirring overnight, then diluted to 0.25 mg mL-1 and filtered with 0.2 µm syringe filters. In the case of spun fibers, ca. 20 mg was used and stirred overnight in 1 mL of the mobile phase, then 100 µL of the liquid phase was diluted to 4 mL of mobile phase. The data was analyzed with the Cirrus GPC Software 3.2.

Table S4. Sample weight in GPC analysis.

Sample for GPC analysis	m (mg)
SKL full	10.0
SKL HMW	10.0
SKL LMW	10.0
WSL	10.0
SKL HMW fiber	23.8
SKL LMW fiber	21.7
WSL fiber	22.1

Lignin chemical structure

1D 1H, and 2D HSQC (Heteronuclear Single Quantum Coherence, hsqcedetgpsisp2.3) and HMBC (Heteronuclear Multiple Bond Correlation, hmbcetgpl3nd) spectra were acquired at 293 K with a Bruker Avance III HD 700 MHz for lignin samples dissolved in 500 µL of DMSO-d_6. 31P NMR spectra were acquired at 293 K with a Bruker Avance III HD 600 MHz to quantify the different hydroxy groups in the lignin samples following the procedure described by Meng *et al.*[2] using cholesterol as the internal standard. All NMR spectra were processed with MestreNova (Version 14.3.3, Mestrelab Research).

Acid insoluble lignin analysis was performed to quantify the lignin content using a two-stage sulfuric acid hydrolysis method following the NREL/ASE standard protocol.[3] Briefly, 100–200 mg of the sample was treated with 72% H_2SO_4 at 30 °C for 60 min. The mixture was diluted to 4% H_2SO_4 and autoclaved at 125 °C for 60 min. After filtration, the collected solid residue was weighed to calculate the weight percent of the acid-insoluble lignin. The acid-soluble lignin in the collected liquor was quantified spectrophotometrically at 205 nm using an extinction coefficient of 110 l $g^{-1}cm^{-1}$.

Thermal analysis

Thermal degradation behavior of the lignins and spun fibers were determined by thermogravimetric analysis (TGA Q5000 IR, TA Instruments, New Castle, DE, USA). Duplicates of 10 mg samples were analyzed. The samples were pre-dried at 105 °C for 20 min and then heated to 700 °C (10 °C min^{-1}) in N2 (25 ml min^{-1}). The onset temperature (T_{onset}) was determined as the temperature at a mass loss of 5 % and char yield (Y_{char}) was the final solid residue at 700 °C normalized against mass at 105 °C. To calculate the mass loss rate, i.e. the first derivative signal (DTG), a numerical differentiation was performed on the thermograms which were smoothed using a 20-points moving average filter.

The glass transition temperature (T_g) was determined with a DSC 1 STARe System (Mettler Toledo). About 5 mg sample was treated in nitrogen flow, 50 ml min^{-1}, in aluminum pans. The moisture was removed in a pre-drying step at 105 °C for 15 min. The temperature was cooled (15 °C min^{-1}) to 25 °C and then the sample was heated to 250 °C (10 °C min^{-1}). The reported T_g is the

average of two replicates determined as the temperature corresponding to the maximum slope of the transition in the DSC curve, obtained from the first derivative of the heat flow.

Mechanical properties of fibers

Tensile testing (Vibroskop/Vibrodyn, Lenzing Instruments, Austria) was performed on conditioned filaments at 20 ± 2 °C and 65 ± 3% RH with an extension rate of 20 mm min^{-1} and a gauge length of 20 mm.

Morphological analysis of cellulose and lignin in spun fibers

Metal replica of wet fibers

Thin metal replicas of wet fibers were prepared using the freeze etching technique called mica sandwich. The wet fibers were cut into shorter lengths and placed on a newly cleaved mica surface together with water, the second sheet of the newly cleaved mica surface was placed on top. The mica sandwich was then plunge frozen in liquid nitrogen followed by separation of the two mica surfaces under liquid nitrogen. The mica sheet was placed in a pre-cooled freeze etching unit (EM ACE900, Leica, Wetzlar, Germany) in which the water was sublimated in vacuum for 2 h at -90 °C. After sublimation of water a thin metal film of platinum, 1 nm, was rotary shadowed on the exposed fibers at a low angle followed by rotary shadowing of carbon, 20 nm, as support for the thin platinum film. The fibers with thin metal replica were further observed in a scanning electron microscope from JEOL, model JSM-7800F. Secondary electron images were acquired using an accelerating voltage of 3 kV and a working distance of 4 mm.

Light microscopy

Light Microscopy was used to analyze the fibers in its wet state (BX53F2 equipped with a CMOS color camera SC50, Olympus, Tokyo, Japan). The fibers were placed on a glass slide with water with a cover glass. The microscopy technique differential interference contrast (DIC) was used which enhances the interfaces.

DIC uses polarized light together with a Nomarski prism which gives differences in path lengths. These differences in path lengths, when passing through the sample, enhance interfaces in the structure by the differences in refractive index, no staining is needed.

Surface and cross sections of dry fibers

Dry fibers were analyzed with SEM using same settings as described for the wet fiber replicas. The fibers were coated with 1.5 nm of platinum. Fibers where also embedded in epoxy, the surface was polished and cross sections were observed using a Sem PhenomProX (Phenom World, Eindhoven, The Netherlands).

Chemical structure lignin in fibers

The dried spun fibers were cut into small pieces and first packed in 4 mm rotors for solid state NMR measurements. After the solid-state NMR measurements, these fibers were dissolved in an electrolyte solution of tetra-n-butylphosphonium acetate $[P_{4444}][OAc]$ diluted with DMSO-d_6 (1:4 wt%) to perform solution NMR measurements.

^{13}C solid-state NMR experiments were carried out on a Bruker Avance III 500 MHz spectrometer equipped with a 4 mm HX CP MAS probe. Cross-polarization (CP) experiments with a contact time of 1.5 ms and a repetition time of 2 s were recorded at a magic angle spinning rate of 10 kHz. The temperature was set to 298 K. 1H decoupling with a "spinal64" scheme at 77 kHz was applied during the acquisition. All CP spectra were recorded with 4000 signal accumulations. The recorded spectra were processed on Topspin 4.2.0 and further processed and normalized with Python 3.12.

The solution NMR experiments were performed on a Bruker Avance III HD 700 equipped with a 5 mm QCI cryoprobe set to 65 °C. A multiplicity edited ^{1}H-^{13}C HSQC (hsqcedetgpsisp2.3) was recorded on the solutions according to the protocol described by Fliri et al.[4] The recorded spectra were processed and analyzed with Topspin 4.2.0.

Lignin leaching during spinning

Ultraviolet-visible (UV/Vis) absorbance (using SPECORDE 200 PLUS, Analytik Jena AG, Jena, Germany) at 280 nm was measured on coagulation baths for determining the amount of leached lignin. The absorbance was converted to lignin concentration based on an extinction coefficient of 24.6 L g^{-1} cm^{-1} (Fengel *et al*. 1981). Correction was made for the contribution to the absorbance from [EMIM]OAc, which was about 1/10th of that of lignin. The [EMIM]OAc concentration in the coagulation bath was determined by conductivity measurements of the baths at 23°C (inoLab Cond 720 Benchtop Conductivity Meter, Thomas Scientific, Swedesboro, NJ, USA) based on a linear calibration curve.

Table S5. Leaching of lignin in the coagulation bath for the different lignins used. The lignin yield is calculated for comparison, based on acid insoluble lignin in prepared solution, assuming all [EMIM]OAc exits the fiber in the coagulation bath (CB).

Type of lignin	g Lignin / g [EMIM]OAc	Lignin Yield (assuming all [EMIM]OAc exits in CB)
SKL	0.0046	94.6%
HMW	0.0007	99.3%
LMW	0.0050	93.0%
Wheat	0.0297	54.1%

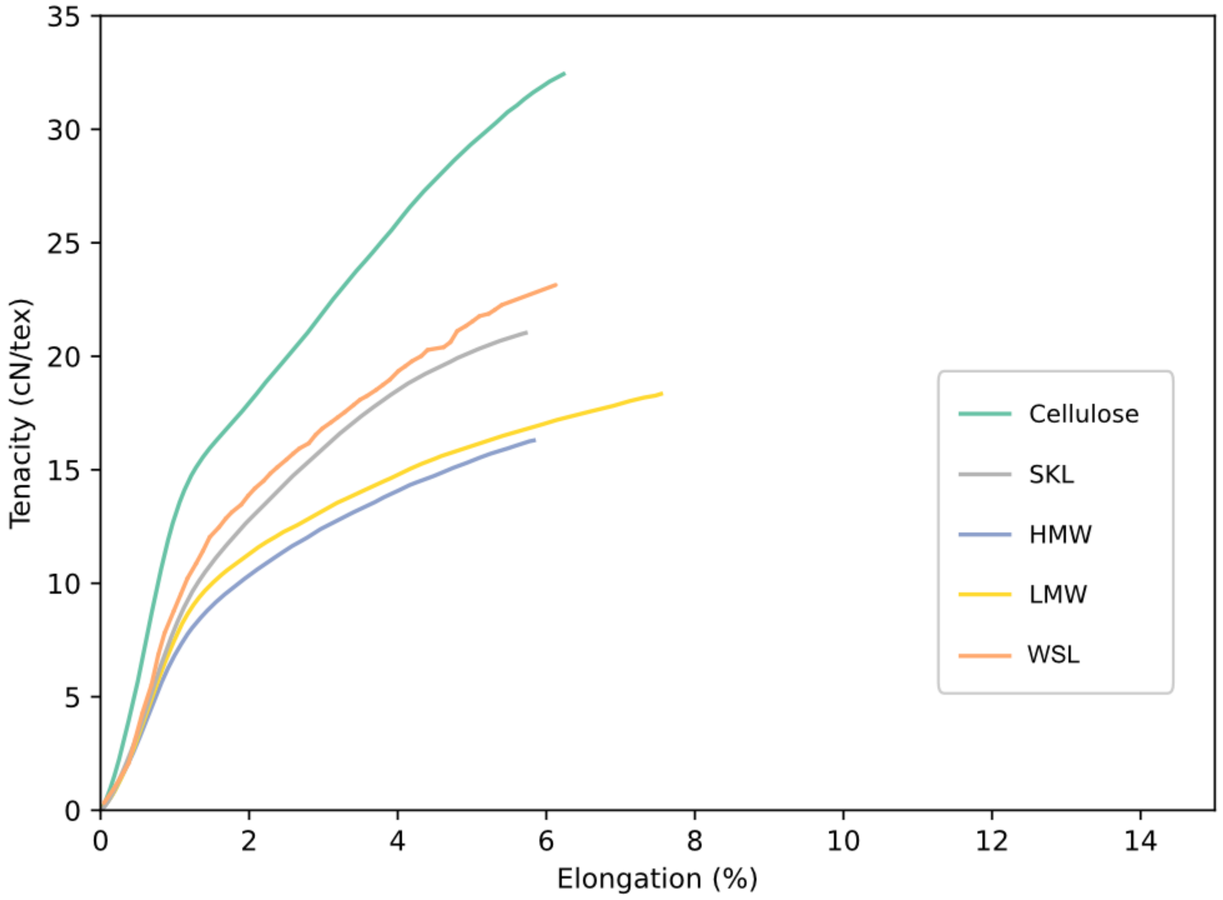

Figure S1. Stress-strain curves of all fibers included in the study. Pure cellulose fibers and lignin-cellulose fibers with SKL, HMW, LMW and WSL lignin.

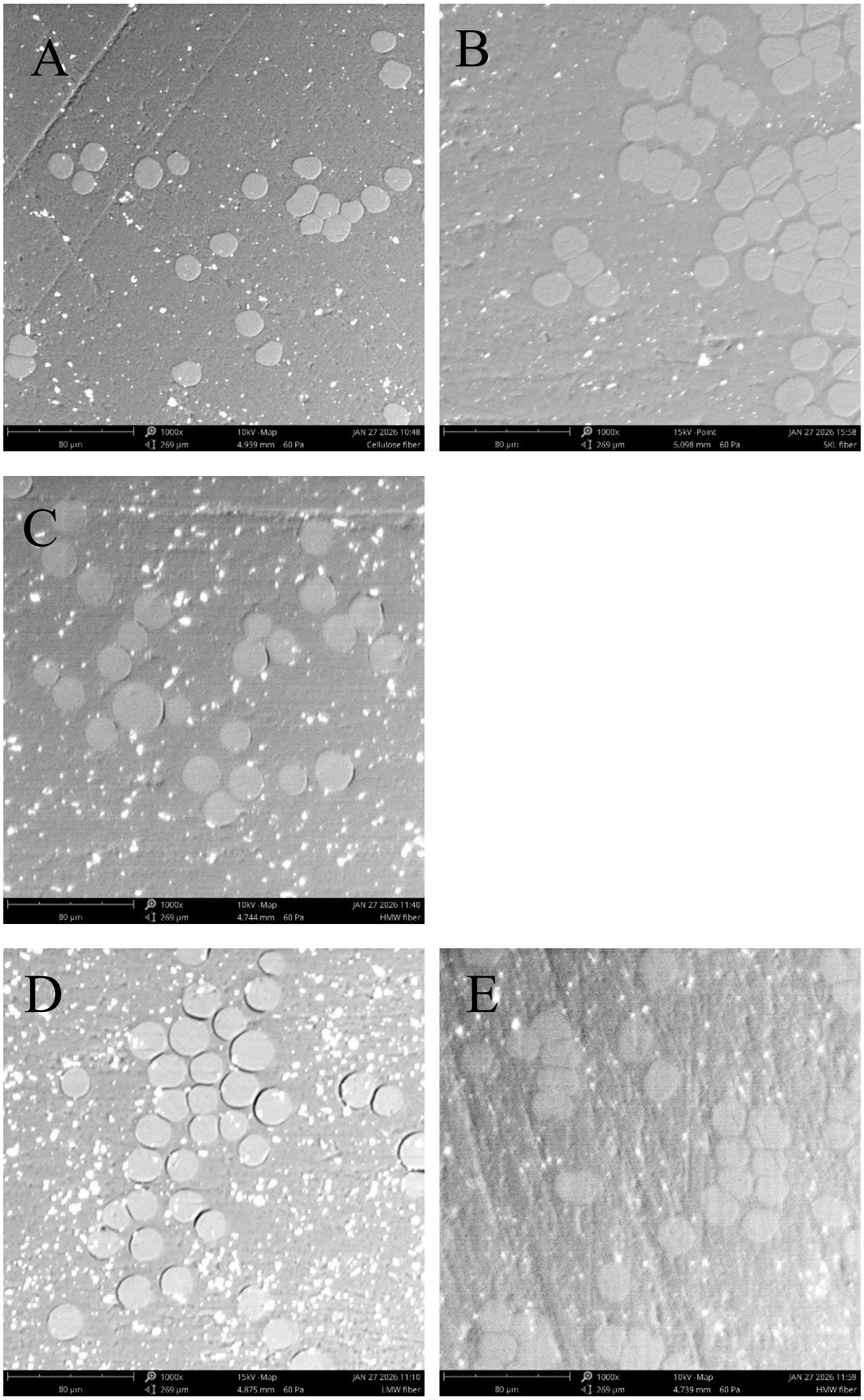

Figure S2. Cross sections of fibers embedded in epoxy, observed in SEM. A. Cellulose, B. SKL-fiber, C. HMW-fiber, D. LMW-fiber, and E. WSL fibers.

a.

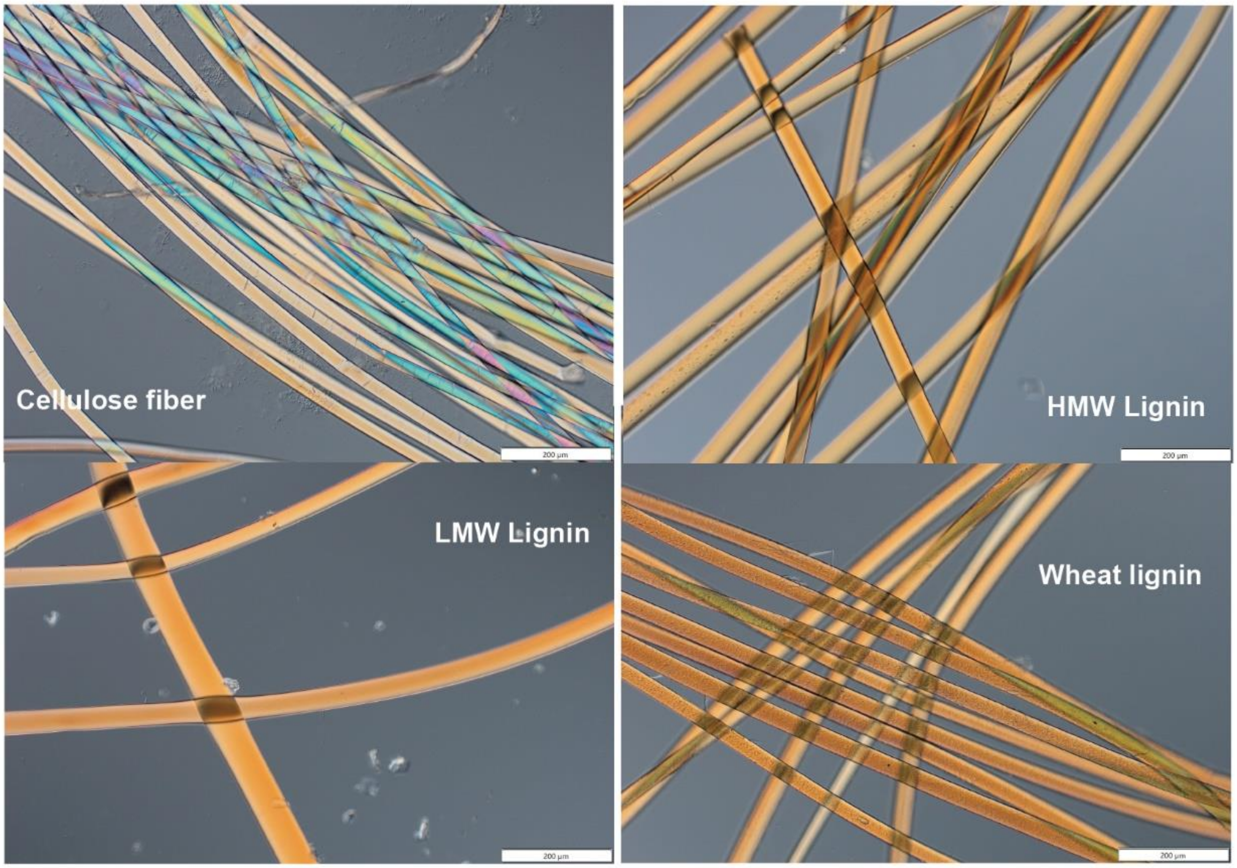

b.

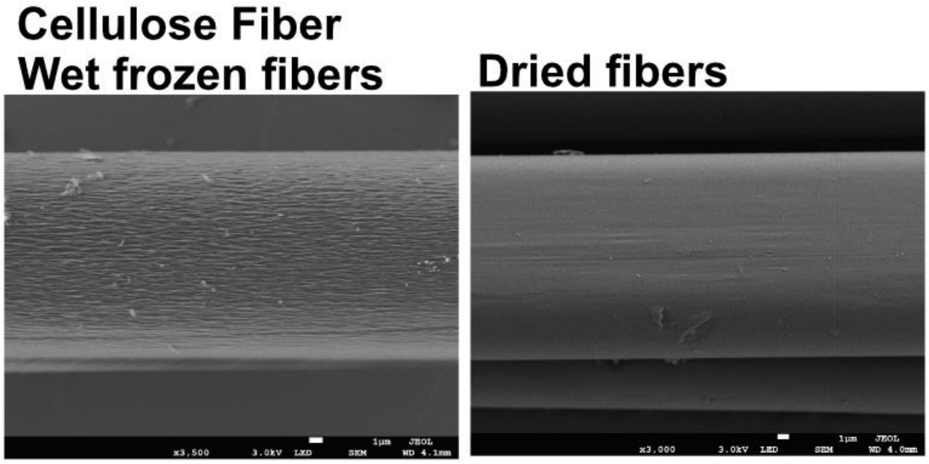

Figure S3. (a) Wet fibers in light microscope. (b) Cellulose fiber replica of wet state and dried fiber.

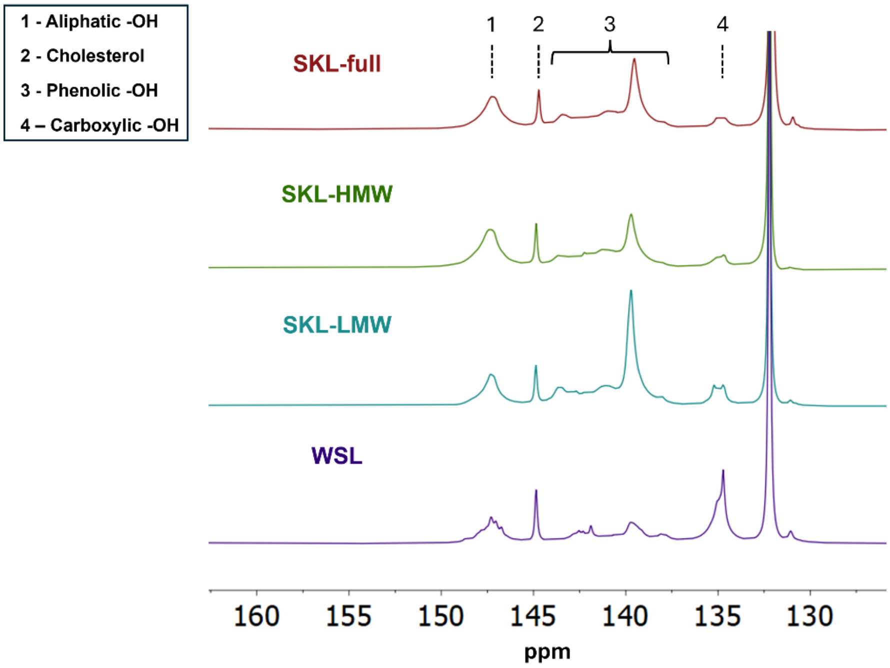

Figure S4. ^{31}P NMR spectra of the four lignin samples.

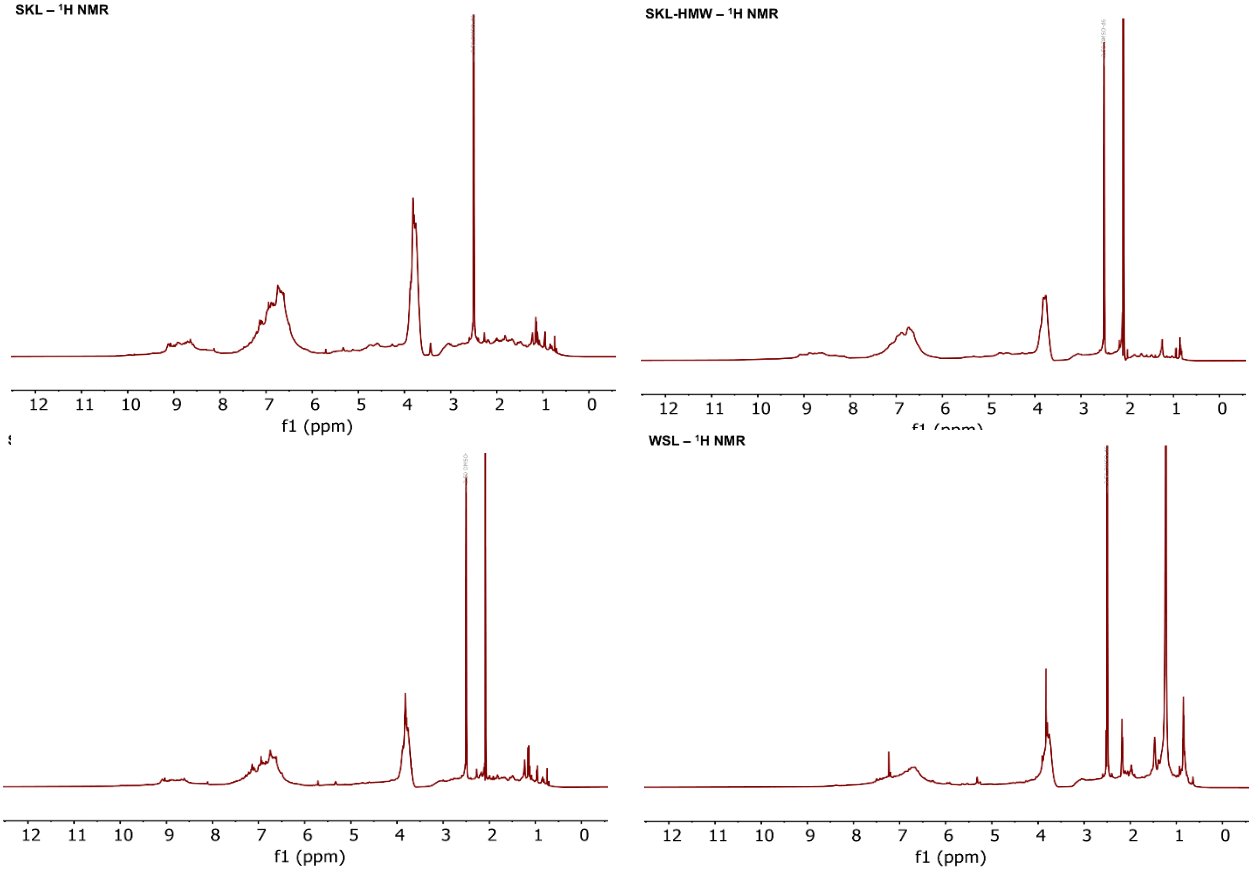

Figure S5. ^{1}H NMR of the four lignin samples.

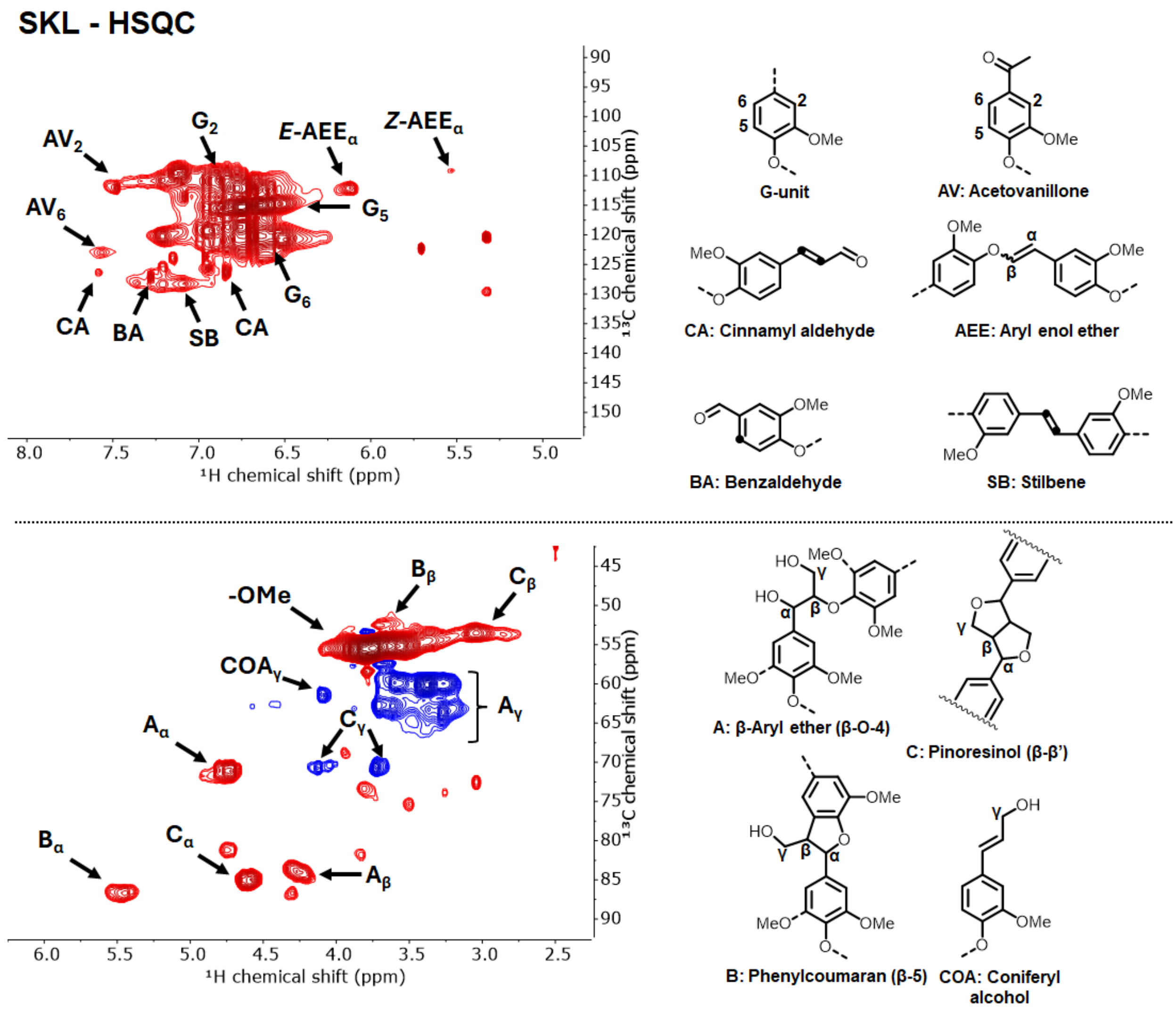

Figure S6. HSQC spectrum of SKL.

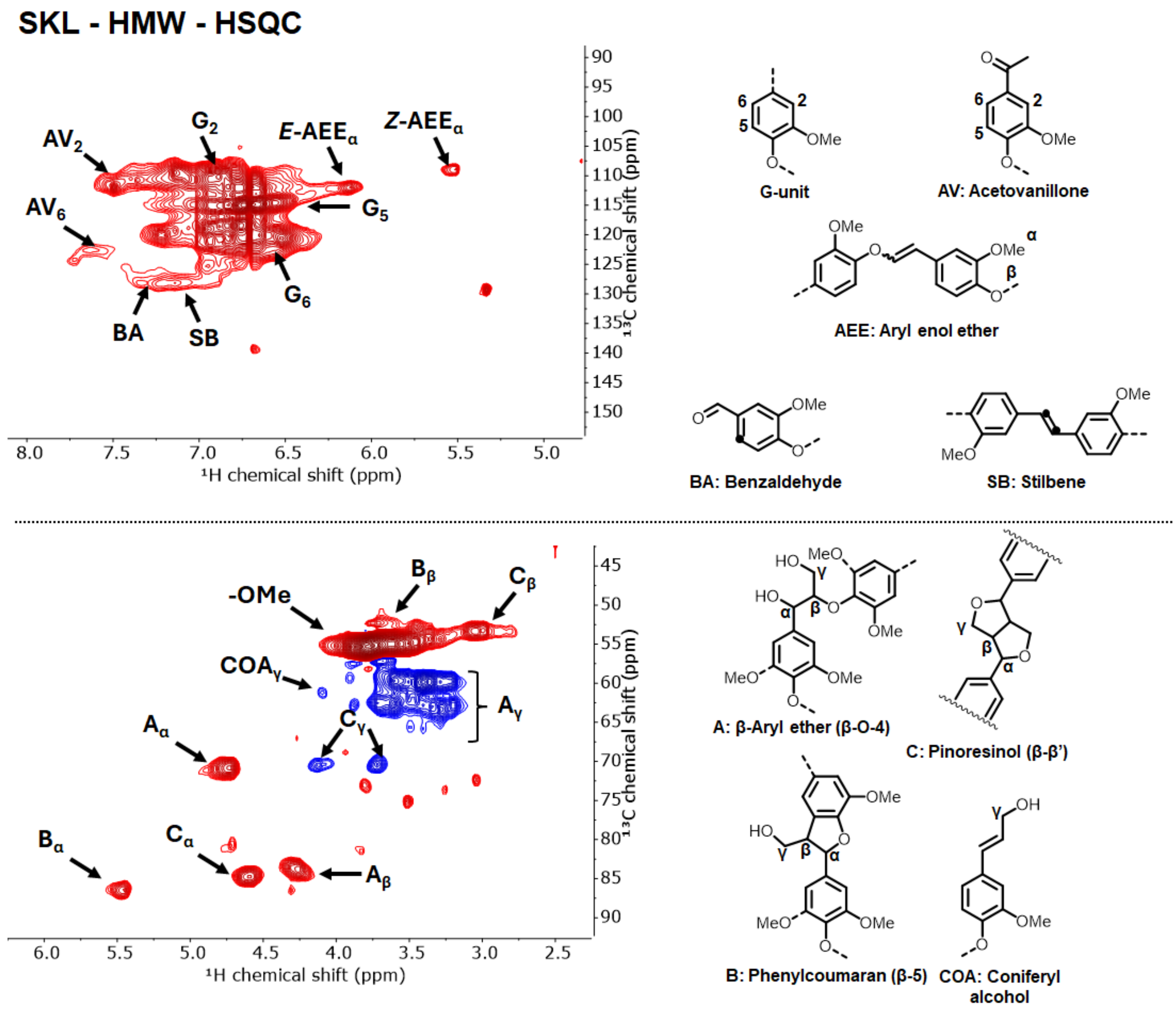

Figure S7. HSQC spectrum of HMW.

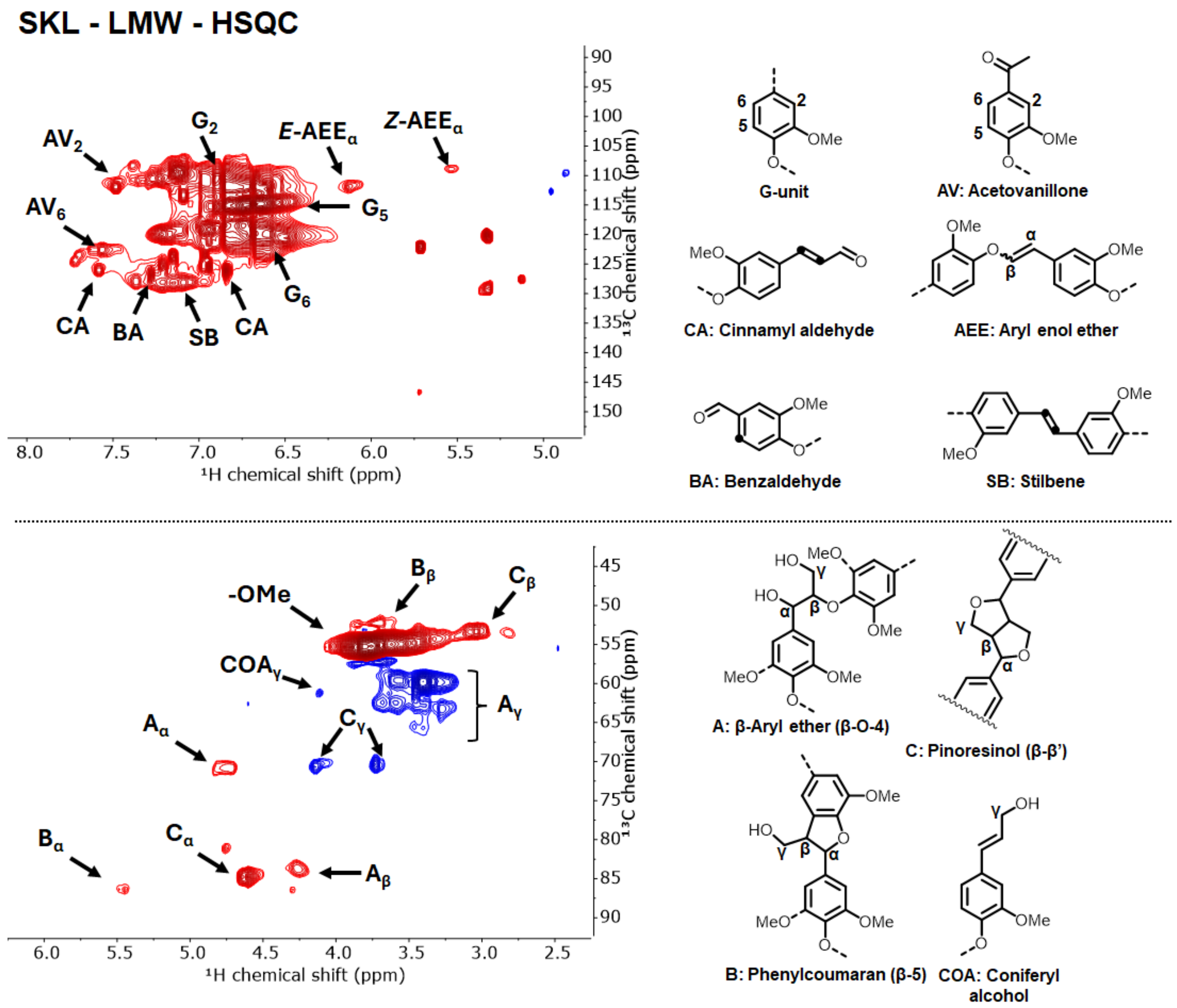

Figure S8. HSQC spectrum of LMW.

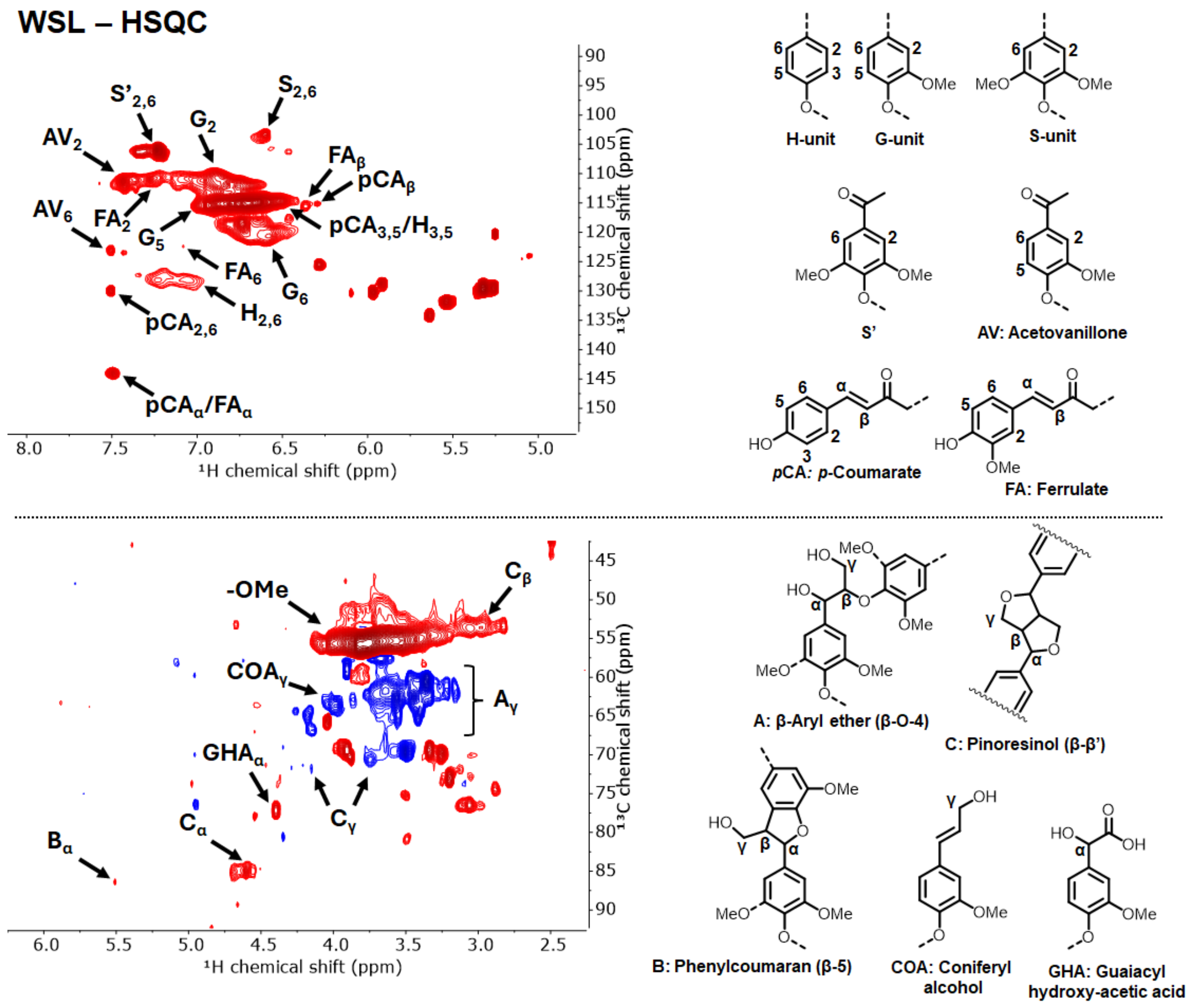

Figure S9. HSQC spectrum of WSL.

Assignments were based on the following references:

- Li, Q. *et al.*[5]
- Crestini, C. *et al.*[6]
- Dräger, H. *et al.* [7]
- Lancefield, C. S. *et al.* [8]

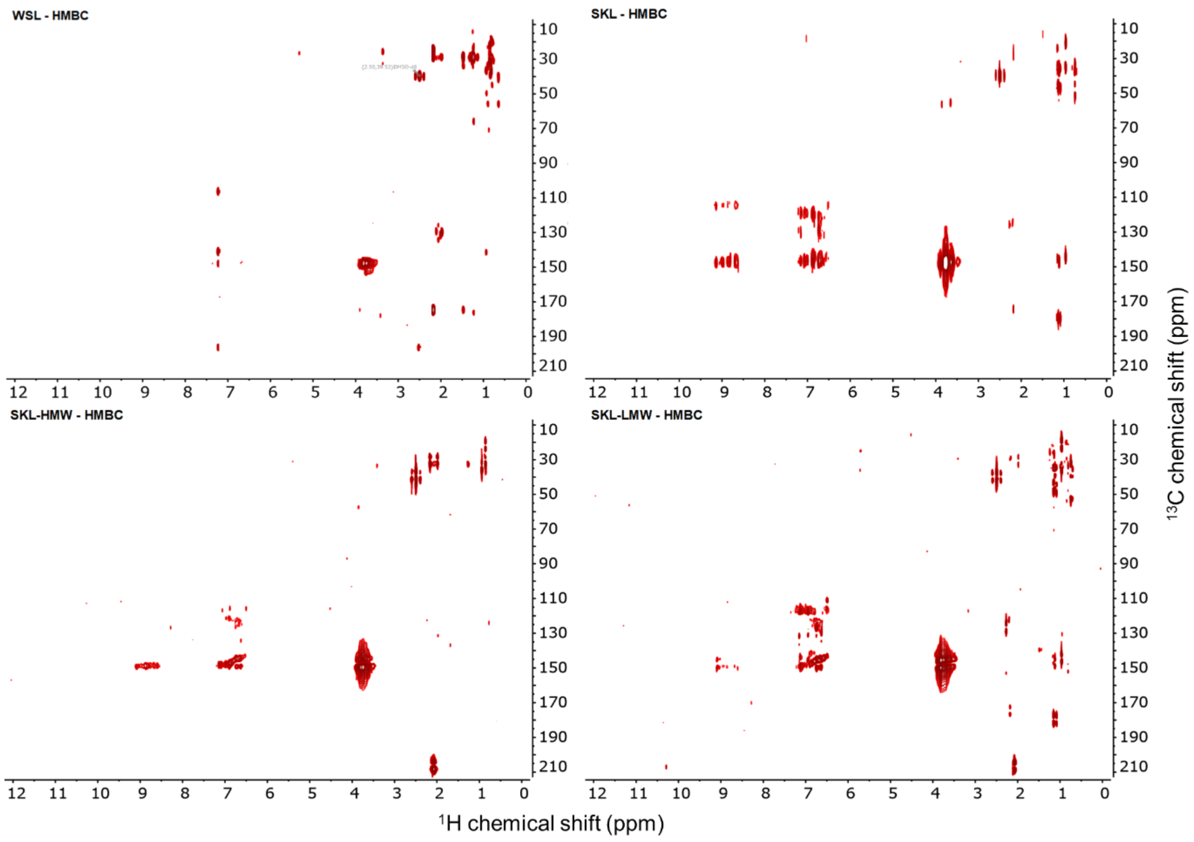

Figure S10. HMBC spectra of the four lignin samples.

Solid state NMR

To investigate the structure of cellulose and lignin in the fibers solid-state ^{13}C CP NMR measurements were performed. The ^{13}C CP MAS spectra of the spun fibers are shown in Figure 4. The recorded spectra delineate the cellulose region in a range of 60 ppm to 110 ppm and the different regions of lignin: aromatic groups at 110–160 ppm, aliphatic groups within the range of 10–50 ppm, and methoxy groups at around 56 ppm. Since solid-state ^{13}C CP NMR spectra are not inherently quantitative, the recorded spectra were normalized by the sum of signal integrals to allow simple comparison and to monitor the lignin leaching.

The main characteristic regions of lignin are visible in the spectra recorded for all the spun fibers. However, for the spun fibers from WSL lignin, the spectra show a slightly different structure and a very low intensity of the lignin regions, which presumably was due to high amounts of lignin leaching.

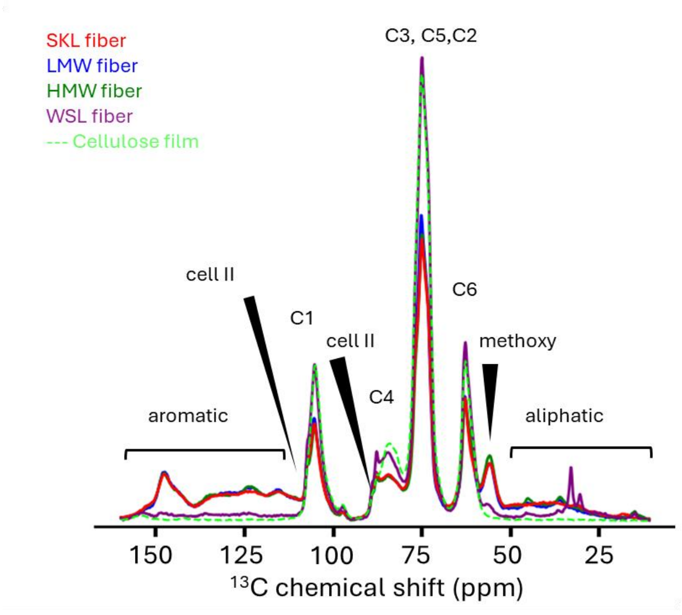

Figure S11. Solid-state ^{13}C CP NMR spectra of spun fibers. The spectra are normalized by the total amount of the signal. A coagulated cellulose film is used as a reference. C refers to cellulose, Cell II to crystalline cellulose II. The numbers refer to the atom number in the molecular structure.

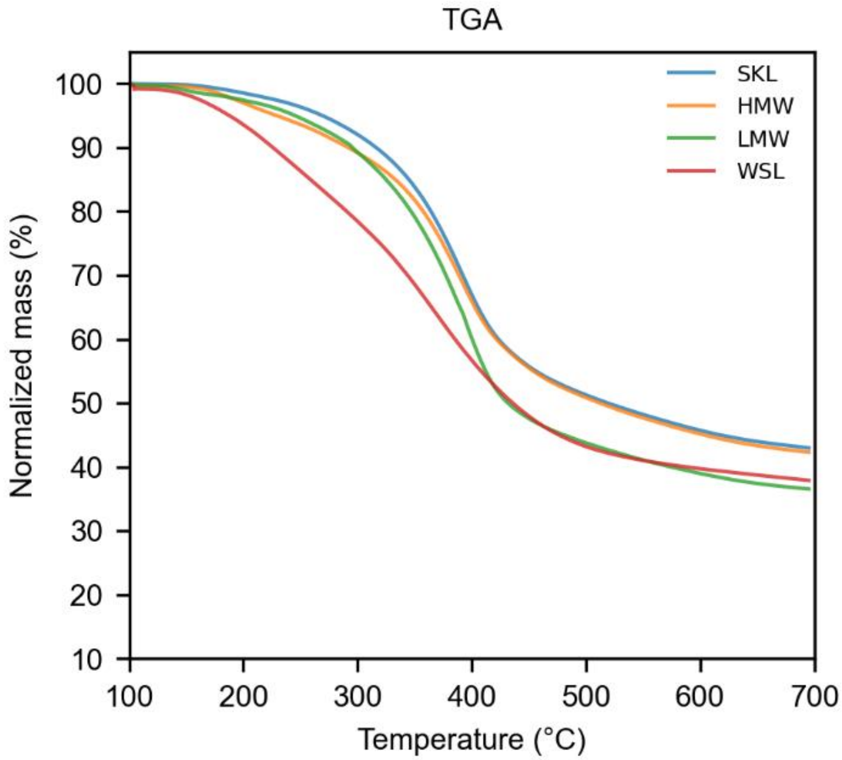

Figure S12. Result of thermo-gravimetrical (TGA) analysis of the used lignins.

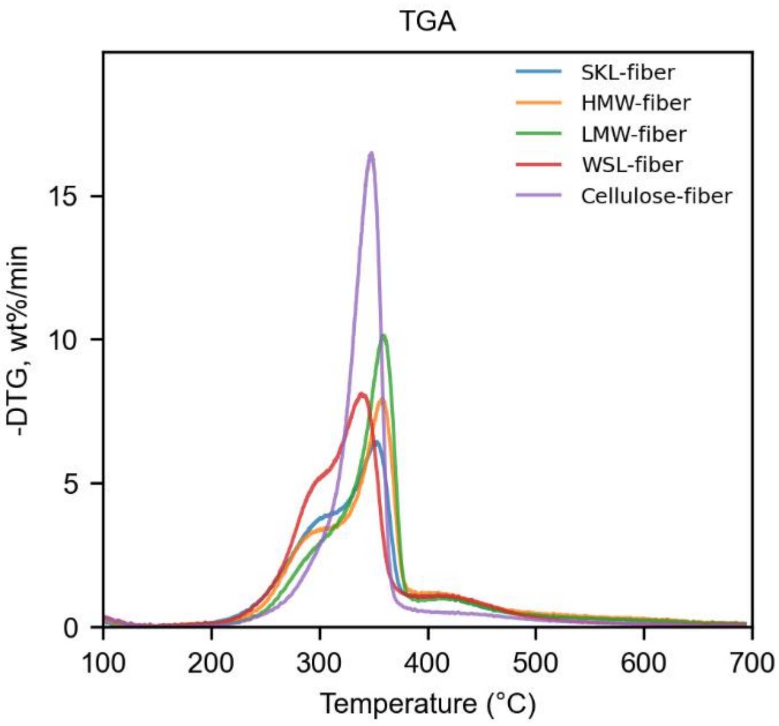

Figure S13. Derivative Thermogravimetric (DTG) curves for spun fibers.

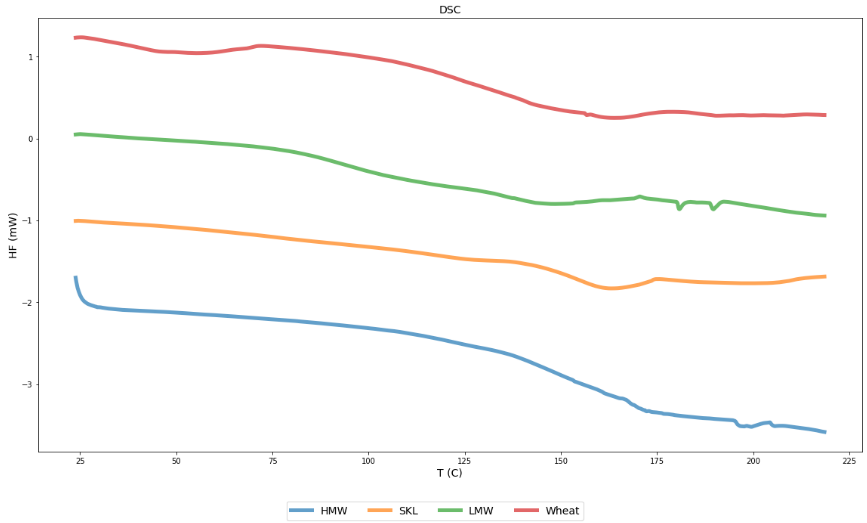

Figure S14. Differential scanning calorimetry (DSC) curves of lignins.

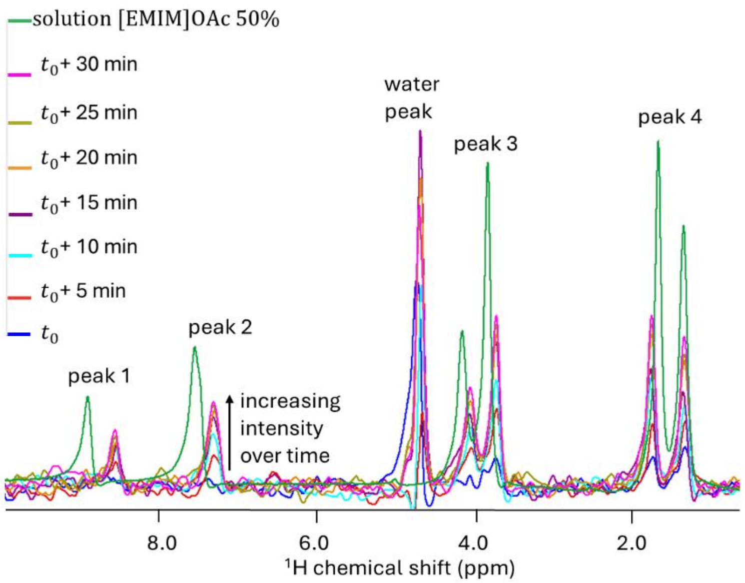

Figure S15. Recorded spectra in a voxel (3 mm^3) during 30 min coagulation of cellulose film in water, using voxel spectroscopic MRI. The [EMIM]OAc peaks 1, 2 and 4 were integrated and used for the evaluation.

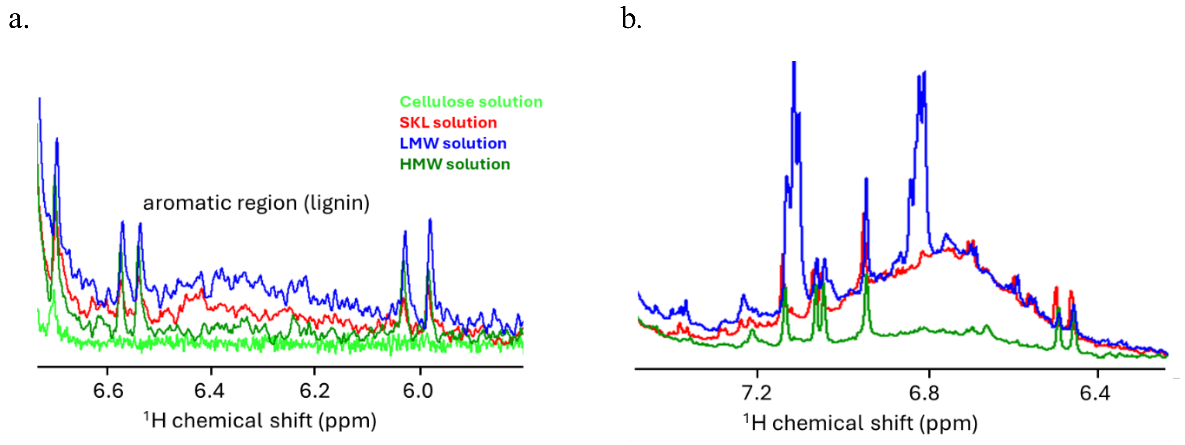

Figure S16. Solution-state NMR analysis of the coagulation baths after 30 min of coagulation of the model films in water (a) and in ethanol (b).

References

(1) Cui, C.; Sun, R.; Argyropoulos, D. S. Fractional Precipitation of Softwood Kraft Lignin: Isolation of Narrow Fractions Common to a Variety of Lignins. *ACS Sustain Chem Eng* **2014**, *2* (4), 959–968. https://doi.org/10.1021/sc400545d.

(2) Meng, X.; Crestini, C.; Ben, H.; Hao, N.; Pu, Y.; Ragauskas, A. J.; Argyropoulos, D. S. Determination of Hydroxyl Groups in Biorefinery Resources via Quantitative 31P NMR

Spectroscopy. *Nat Protoc* **2019**, *14* (9), 2627–2647. https://doi.org/10.1038/S41596-019-0191-1;SUBJMETA.

(3) Sluiter, A.; Hames, B.; Hyman, D.; Payne, C.; Ruiz, R.; Scarlata, C.; Sluiter, J.; Templeton, D.; Wolfe, J. *Determination of Total Solids in Biomass and Total Dissolved Solids in Liquid Process Samples, Laboratory Analytical Procedure (LAP)*; 2008.

(4) Fliri, L.; Heise, K.; Koso, T.; Todorov, A. R.; Rico del Cerro, D.; Hietala, S.; Fiskari, J.; Kilpeläinen, I.; Hummel, M.; King, A. W. T. Solution-State Nuclear Magnetic Resonance Spectroscopy of Crystalline Cellulosic Materials Using a Direct Dissolution Ionic Liquid Electrolyte. *Nat Protoc* **2023**, *18*, 2084–2123.

(5) Li, Q.; Xie, S.; Serem, W. K.; Naik, M.; Yuan, J. S. Quality Carbon Fibers from Fractionated Lignin. *Green Chemistry* **2017**, *19*, 1628–1634.

(6) Crestini, C.; Lange, H.; Sette, M.; Argyropoulos, D. S. On the Structure of Softwood Kraft Lignin. *Green Chemistry* **2017**, *19*, 4104–4121. https://doi.org/10.1039/c7gc01812f.

(7) Dräger, H.; Mobley, J.; Kamali, P.; Dorrani, M.; Lynn, B.; DeHaan, L.; Schendel, R. R. Lignin, Extractives and Structural Carbohydrate Characteristics of Thinopyrum Intermedium Biomass Reveal Additional Valorization Opportunities for Dual-Crop Utilization. *J Sci Food Agric* **2024**, *104* (15), 9451–9461. https://doi.org/10.1002/JSFA.13768;WEBSITE:WEBSITE:SCIJOURNALS;PAGE:STRING:ARTICLE/CHAPTER.

(8) Lancefield, C. S.; Wienk, H. J.; Boelens, R.; Weckhuysen, B. M.; Bruijnincx, P. C. A. Identification of a Diagnostic Structural Motif Reveals a New Reaction Intermediate and Condensation Pathway in Kraft Lignin Formation. *Chem Sci* **2018**, *9* (30), 6348–6360. https://doi.org/10.1039/C8SC02000K.
